# The Potential of the Adzuki Bean (*Vigna angularis*) and Its Bioactive Compounds in Managing Type 2 Diabetes and Glucose Metabolism: A Narrative Review

**DOI:** 10.3390/nu16020329

**Published:** 2024-01-22

**Authors:** Shu Hang Kwan, Elvira Gonzalez de Mejia

**Affiliations:** 1 Division of Nutritional Sciences, University of Illinois at Urbana-Champaign, Champaign, IL 61801, USA; shuhk2@illinois.edu; 2 Department of Food Science and Human Nutrition, University of Illinois at Urbana-Champaign, Champaign, IL 61801, USA

**Keywords:** adzuki beans, *Vigna angularis*, type 2 diabetes, prevention, management, glucose metabolism, obesity, dyslipidemia, gut microbiota, oxidative stress

## Abstract

Type 2 diabetes (T2D) is a common noncommunicable disease. In the United States alone, 37 million Americans had diabetes in 2017. The adzuki bean (*Vigna angularis*), a legume, has been reported to possess antidiabetic benefits. However, the extent and specific mechanisms through which adzuki bean consumption may contribute to T2D prevention and management remain unclear. Therefore, the aim of this narrative review is to analyze current evidence supporting the utilization of adzuki beans in the diet as a strategy for preventing and managing T2D. Animal studies have demonstrated a positive impact of adzuki beans on managing T2D. However, supporting data from humans are limited. Conversely, the potential of adzuki bean consumption in preventing T2D via modulating two T2D risk factors (obesity and dyslipidemia) also lacks conclusive evidence. Animal studies have suggested an inconsistent and even contradictory relationship between adzuki bean consumption and the management of obesity and dyslipidemia, in which both positive and negative relationships are reported. In sum, based on the existing scientific literature, this review found that the effects of adzuki bean consumption on preventing and managing T2D in humans remain undetermined. Consequently, human randomized controlled trials are needed to elucidate the potential benefits of the adzuki bean and its bioactive components in the prevention and management of T2D.

## 1. Introduction

According to the World Health Organization, diabetes is one of the four most common non-communicable diseases in the world, and it accounts for two million non-communicable deaths annually [1]. In the United States, more than 37 million Americans have diabetes; 90–95% of them have type 2 diabetes (T2D) [2]. In 2017, the total cost of medical care for diagnosed diabetes and loss of productivity for diabetics in the United States was USD 327 billion, and about 25% of the healthcare spending was on people with diagnosed diabetes [3]. Likewise, diabetes poses a significant challenge in Europe. *The International Diabetes Federation Diabetes Atlas* reported that 61 million Europeans suffered from diabetes in 2021 and it caused 1.1 million deaths [4]. Therefore, it is important to develop alternative means to prevent and manage this disease.

T2D is an intricate metabolic condition. It is distinguished by persistent high blood glucose levels, which result in microvascular and macrovascular complications [5]. According to the American Diabetes Association Standards of Medical Care in Diabetes-2022, the criteria for its diagnosis is a fasting plasma glucose ≥ 126 mg/dL (7 mmol/L), or a 2-h plasma glucose value ≥ 200 mg/dL (11.1 mmol/L) during a 75 g oral glucose tolerance test, or a Hemoglobin A1C (HbA1c) ≥ 6.5% (48 mmol/mol) [6]. Neuropathy, nephropathy, retinopathy, cardiovascular disease, stroke, and peripheral vascular disease are common complications experienced by patients with T2D [7,8]. Diabetes can speed up the advancement of liver damage, and disrupt glucose and lipid metabolism [9].

The liver has a central role in managing carbohydrate and fat metabolism, which is important for maintaining insulin sensitivity [10]. Increased hepatic gluconeogenesis contributes to diabetic hyperglycemia [11]. Moreover, some of the main contributors to T2D are chronic insulin resistance and the progressive loss of β-pancreatic cell insulin secretion [5,12,13]. Kim et al. [14] reported that prolonged exposure to high glucose levels resulted in lowered pancreatic cell viability and impaired insulin secretion. In particular, the phosphoinositide 3-kinases/protein kinase B (PI3K/AKT) signaling pathway was found to contribute to the transduction of insulin signaling and the regulation of glucose metabolism [15,16]. In addition to abnormal glucose metabolism, obesity also contributes to T2D [17]. Obesity causes dysregulation of pro-inflammatory adipokines and anti-inflammatory adipokines, leading to the development of chronic inflammation and insulin resistance and resulting in T2D [18].

Although various medications are available for managing T2D, lifestyle modifications, such as weight loss and diet modifications, also play a part in T2D management [19]. It has been shown that plant proteins are beneficial for managing diabetes. According to a meta-analysis of 15 randomized controlled trials, substituting animal protein with plant protein from legumes including several pulses such as beans, peas, chickpea, lentils and soybean improved glycemic control in individuals with diabetes [20]. Legume consumption is recommended as part of the traditional plant-based diets by several organizations, such as the American Heart Association Nutrition Committee and Diabetes of Canada, due to their nutritional value and diverse potential health benefits [21,22]. A randomized control trial found that consuming one cup of legume per day for three months significantly lowered the HbA1c of the participants [23]. Legume proteins and phenolic compounds were found to contribute to the benefits associated with bean consumption [24,25,26]. Legumes are one of the recommended food items for diabetic diets, as they have a low glycemic index. According to Atkinson et al. [27], 94% of the legumes they investigated were considered food with a low glycemic index, 6% of the legumes were considered food with a medium glycemic index, and none of the legumes were considered food with a high glycemic index. A randomized controlled trial found that increased legume consumption might prevent T2D [28]. The adzuki bean (*Vigna angularis*) is a legume that has a low glycemic index of 26 and is one of the beans used in Asian cultures to improve T2D [29]. Therefore, it has the potential to serve as an alternative to manage and prevent T2D. Yet, the extent and specific mechanisms through which adzuki bean consumption contributes to the prevention and management of T2D remain unclear. Therefore, this review will discuss current evidence supporting the use of adzuki bean supplements as a means to prevent and manage T2D. The primary objective of this review was to assess the possibility of using adzuki bean consumption as an alternative strategy to manage T2D and determine the underlying mechanism as it relates to insulin signaling and glucose metabolism. In addition, it also explores the prospects that adzuki bean supplementation holds for T2D prevention by assessing two key diabetes risk factors: obesity and dyslipidemia.

## 2. Methods

This narrative review used the PubMed and Scopus databases and searched the keywords “adzuki bean” and “diabetes”, and “adzuki bean” and “obesity”. Human studies, animal studies, and cell culture studies published were included. Studies that were not published in English were excluded. Results were included if adzuki bean consumption was used to manage T2D, obesity, and dyslipidemia outcomes.

## 3. Background for Adzuki Beans

**Figure 1:**
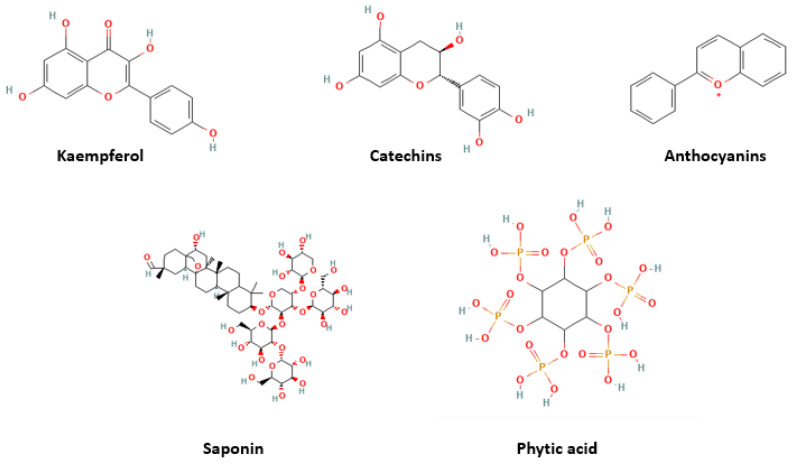
Chemical structures of some of the adzuki bean bioactive compounds, including kaempferol, catechins, anthocyanins, saponin, and phytic acid. Protein and peptides are not highlighted in this figure.

The adzuki bean (*Vigna angularis*) is one of the 12 most important legumes in Asia. It is cultivated in more than 30 countries, with China being the largest producer [30,31]. Various cultures process the adzuki bean differently. The adzuki bean is consumed as a sweetened bean paste in Japan [31]. In other countries where people consume adzuki beans daily, they are consumed as whole beans [32]. The bean is also processed into porridge, bean paste, and soup; however, it is not consumed raw, and needs to be cooked before consumption. In Western culture, the adzuki bean is added to soups, stews, and salads [33]. Table 1 presents examples of commercially available food products that contain adzuki beans. Although research findings suggest potential anti-diabetic health benefits of adzuki bean supplementation, few functional food products contain or are derived from adzuki beans in the current market. Adzuki beans are used as part of the ingredients in commercially available food products, such as ready-to-eat meals, desserts, confectionery, pastries, snacks, and beverages (Table 1). However, there is significantly less variety in food options based on adzuki beans compared to other legumes, such as chickpeas. Moreover, most of the adzuki bean commercial food products are made in Japan. Therefore, adzuki bean functional food development using various food processing technologies presents great business potential for the food industry. In China, these beans have been used since the Tang Dynasty (618–907 AD) as medical treatments, for conditions such as diuresis, swelling, abscesses, and weight control [34]. The bioactive compounds in the adzuki bean might contribute to its potential health benefits. Figure 1 presents some of the most important bioactive compounds found in the adzuki bean; of course, proteins and peptides are also important to consider. But limited research has been conducted to identify the bioactive peptides in the adzuki bean. Furthermore, studies have also reported that the adzuki bean exhibits antidiabetic benefits [35,36]. For example, adzuki bean polysaccharides exhibited antidiabetic effects via the insulin-PI3K-AKT signaling pathway, which contributes to the transduction of insulin signaling and the regulation of glucose metabolism, in T2D rats [15,16,37]. Therefore, adzuki bean consumption deserves in-depth research to better understand the underlying mechanism of action associated with its health benefits.

## 4. The Role of Adzuki Bean Supplements in Managing T2D Outcomes

Human and animal studies have suggested that the consumption of adzuki bean has various health benefits, including antidiabetic effects. To date, only one human study, thirteen animal studies, and two in vitro studies have investigated the antidiabetic potential of the adzuki bean (Table 2). The research on the anti-diabetic properties of the bean has been focused on their bioactive compositions, including polyphenols, polysaccharides, and peptides/proteins. As indicated in Table 2, out of 13 animal studies, 13 showed that experimental animals who received adzuki bean treatments revealed improvements in T2D indicators such as blood glucose levels, insulin sensitivity, and glucose tolerance. The human study showed that subjects who received extruded adzuki bean as convenience food had similar improvement in T2D indicators as subjects who received a traditional diabetic low glycemic index diet [29]. These data support the notion that adzuki bean consumption could be beneficial in managing T2D.

### 4.1. Effectiveness of Adzuki Bean Flour on T2D Outcomes

Hyperglycemia is one of the T2D-related outcomes for studying the effectiveness of diabetes management. High-fat diets have been found to contribute to higher blood glucose levels, and various studies have used high-fat diets as one of the means to induce high blood glucose in mice [10,36,37,38,39]. All these investigations found that supplementing high-fat diets with adzuki bean flour could improve such conditions. Zhao et al. [36] found that providing 4-week-old male C57BL/6 mice with a high-fat diet consisting of 60% kcal from fat significantly increased their fasting blood glucose, fasting serum insulin, and insulin resistance (*p* < 0.01). However, supplementing the high-fat diet with 150 mg adzuki bean flour/kg diet/day for 12 weeks significantly decreased fasting blood glucose (*p* < 0.05), fasting serum insulin (*p* < 0.05), the HOMA-IR index (*p* < 0.01), and blood glucose levels during an oral glucose tolerance test (*p* < 0.01) [36]. Another study revealed similar improvements in T2D-related outcomes in streptozotocin-induced diabetic mice. Diabetic male C57BL/6J mice had significantly higher fasting blood glucose levels and glycated serum protein levels than healthy non-diabetic mice; however, after providing these diabetic mice with 300 g raw adzuki bean flour/kg diet for 8 weeks, their fasting blood glucose and glycated serum protein levels decreased significantly (*p* < 0.05) [10]. Moreover, the researchers also found that diabetic mice had a significantly higher area under the curve for the oral glucose tolerance test, while the area under the curve for the oral glucose tolerance test was significantly lower in diabetic mice that received 300 g raw adzuki bean flour/kg body weight (BW) (*p* < 0.05). These results indicate that in diabetic mice, consuming adzuki bean flour improves glucose tolerance.

### 4.2. Effectiveness of Adzuki Bean Polyphenols on T2D Outcomes

Polyphenols in adzuki beans also play a positive role in regulating T2D outcomes, especially in lowering blood glucose levels. Itoh et al. [41,42] observed that providing adzuki bean hot water extracts (obtained by boiling adzuki beans in water to extract the polyphenols) to male KK-A^y^ mice with T2D significantly lowered their blood glucose levels. Compared to their control counterparts that received 500 mg of cellulose/kg of body weight/day, the mice receiving 500 mg of the hot water extract showed significantly lower blood glucose at weeks two, three, five, and six after the administration of the extract-supplemented diet (*p* < 0.05) [41]. Larger antidiabetic effects were exhibited in diabetic mice receiving a higher dose of adzuki bean extract. Diabetic mice receiving 5000 mg hot water extract from adzuki beans/kg BW/day had significantly lower blood glucose from weeks one to seven (*p* < 0.05). The diabetic mice receiving 5000 mg hot water extract from adzuki beans/kg BW/day also had lower blood glucose levels than diabetic mice receiving 500 mg hot water extract from adzuki beans/kg BW/day. Moreover, after receiving 5000 mg hot water extract from adzuki beans/kg BW/day for seven weeks, the mice had a significantly lower plasma insulin concentration than mice receiving 5000 mg of cellulose/kg BW/day (*p* < 0.05). On the other hand, diabetic mice receiving 500 mg of hot water extract from adzuki beans/kg BW/day had no significant difference in plasma insulin compared to mice receiving 500 mg of cellulose/kg BW/day (*p* > 0.05). However, diabetic mice receiving 500 mg or 5000 mg hot water extract did not show a statistically significant reduction in HbA1c levels compared to their counterparts that received cellulose supplementation. In another study, Itoh et al. [42] investigated the impact of the hot water extract from adzuki beans on postprandial blood glucose levels. They reported that adzuki bean extract showed some potential in reducing postprandial glucose levels after sucrose administration. Diabetic rats that received 500 mg hot water extract from adzuki beans/kg BW had significantly lower postprandial blood glucose than diabetic rats that received the hypoglycemic agent sulfonylurea 30 and 60 min after the sucrose administration (*p* < 0.05). However, when providing diabetic rats with glucose and soluble starch, there was not a significant difference in postprandial glucose levels between diabetic rats that received hot water extract from adzuki beans and those that did not (*p* > 0.05). Therefore, more studies are needed to examine the effect of adzuki bean polyphenols on postprandial blood glucose levels and the levels of HbA1c.

### 4.3. Effectiveness of Adzuki Bean Polysaccharides on T2D Outcomes

Adzuki bean polysaccharides have been found to help manage T2D by improving glucose homeostasis and pancreatic islet cell recovery in rodents [37,40]. Streptozotocin-induced diabetic Kunming mice were found to have a significantly lower (*p* < 0.05) hepatic glycogen content than the healthy nondiabetic mice; however, treating the diabetic mice with 400 mg/kg BW of adzuki bean polysaccharides for four weeks significantly increased their hepatic glycogen content [40]. These diabetic mice also had significantly higher fasting blood glucose levels than the healthy nondiabetic mice (*p* < 0.01). However, after treating the diabetic mice with adzuki bean polysaccharides for three and four weeks, there was a significant decrease in their fasting blood glucose levels (three weeks: *p* < 0.05; four weeks: *p* < 0.01). In addition, the diabetic mice had a significantly lower insulin sensitivity index than the nondiabetic mice (*p* < 0.01), and this index increased significantly after treating the diabetic mice with adzuki bean polysaccharides for four weeks (*p* < 0.05). This observation suggested the potential of adzuki bean polysaccharides for improving insulin resistance. Adzuki bean polysaccharides also contributed to the recovery of pancreatic cells. In the pancreas of the diabetic mice, there was a decrease in the number of islet cells; however, after providing the diabetic mice with adzuki bean polysaccharides for four weeks, an increased islet cell number was observed. Another animal study also showed dose-dependent antidiabetic effects of adzuki bean polysaccharides [37]. Streptozotocin-induced diabetic male Wistar rats treated with 200 mg/kg BW/day and 400 mg/kg body weight/day of adzuki bean polysaccharides for four weeks had a significantly lower fasting blood glucose level than the diabetic rats on days 14, 21, and 28 (*p* < 0.05). Diabetic rats treated with 100 mg/kg BW/day of adzuki bean polysaccharides for four weeks had significantly lower fasting blood glucose than the diabetic rats on day 28 (*p* < 0.05). Diabetic rats treated with 400 mg/kg BW/day of adzuki bean polysaccharides had an improvement in glucose tolerance, as they had a significantly smaller area under the curve for the oral glucose tolerance test than the diabetic rats (*p* < 0.05). Furthermore, diabetic rats treated with various dosages of adzuki bean polysaccharide had a significantly lower fasting serum insulin level and a significantly higher insulin sensitivity than the diabetic rats (*p* < 0.05) suggesting a decrease in insulin resistance and better glucose metabolism. These benefits were dose-dependent, as higher adzuki bean polysaccharide dosages exhibited better antidiabetic effects.

### 4.4. Effectiveness of Extruded Adzuki Bean Proteins on T2D Outcomes

Extruded adzuki bean proteins had the potential, in mice, to reduce glucose levels, and the antidiabetic effects were dose-dependent [44,45]. An animal study using male diabetic KK-A^y^ mice found that a 2% extruded adzuki bean protein consumption was more effective than no extruded adzuki bean protein consumption in reducing blood glucose levels in diabetic mice [44]. Diabetic mice receiving 2% extruded adzuki bean protein had significantly lower blood glucose than the diabetic mice in the control group (with no adzuki bean protein consumption) starting from week one (*p* < 0.05). On the other hand, diabetic mice receiving 1% extruded adzuki bean protein had significantly lower blood glucose than the diabetic mice in the control group starting at week two (*p* < 0.05), which is one week after the diabetic mice that received 2% extruded adzuki bean protein. In addition, after consuming extruded adzuki bean protein for 6 weeks, mice were found to have a better glucose tolerance. After 30 min of 2 g glucose/kg BW administration after fasting, diabetic mice receiving extruded adzuki bean protein had a significantly lower blood glucose level than the diabetic mice in the control group (*p* < 0.05). Also, mice receiving 2% extruded adzuki bean protein had a lower blood glucose level than mice receiving 1% extruded adzuki bean protein, suggesting a better glucose tolerance in mice receiving a higher dosage of extruded adzuki bean protein. Zhao et al. [45] found that, compared to mice that consumed only a high-fat diet, the fasting blood glucose and fasting serum insulin in mice consuming both the heat-treated (boiled for 10 min in water) adzuki bean protein hydrolysates and high-fat diet for 12 weeks were significantly lower (fasting blood glucose: *p* < 0.01; fasting serum insulin: *p* < 0.05). Also, mice consuming both the heat-treated adzuki bean protein hydrolysates and high-fat diet had improved insulin and glucose tolerance, as evidenced by significant decreases in the HOMA-IR index and the area under the curve for the oral glucose tolerance test (*p* < 0.01). Therefore, studies investigating the relationship between various adzuki bean protein supplementary dosages and their effectiveness in managing blood glucose levels are needed to determine the optimal adzuki bean supplementation levels for managing T2D outcomes.

### 4.5. The Potential for Modifying the Antidiabetic Capacity of Adzuki Bean Supplements via Different Food Processing Methods

Adzuki bean supplements have beneficial effects on managing T2D-related outcomes, and different food processing methods could potentially modify the antidiabetic capacity of its bioactive compounds. Food processing contributes to variation in the content of the bioactive compounds, and thus their potential anti-diabetic benefits.

#### 4.5.1. Effectiveness of Cooking on the Antidiabetic Capacity of Adzuki Bean Supplements

Zhao et al. [10] observed that the percentage of flavonoids and total catechins of two adzuki bean bioactive compounds in cooked adzuki beans (12 h soaking at room temperature, 2 h steaming in a steam chamber, and 12 h drying at 40 °C in an oven) were significantly lower than that in the raw adzuki beans. While there was a significant decrease (*p* < 0.05) in glycated serum protein in diabetic mice that consumed either 300 g raw adzuki bean flour/kg BW or 300 g cooked adzuki bean flour/kg BW for eight weeks, cooked and raw adzuki bean flour had different effects on various diabetic-related outcomes. Compared to the diabetic counterparts that did not receive any adzuki bean flour, diabetic mice consuming 300 g raw adzuki bean flour/kg BW for eight weeks had significantly lower fasting blood glucose levels and significantly higher fasting serum insulin levels (*p* < 0.05) [10]. However, no significant changes were found in the fasting blood glucose and fasting serum insulin levels in diabetic mice consuming 300 g cooked adzuki bean flour/kg BW for eight weeks (*p* > 0.05). Food processing also impacted the degree of glucose tolerance in diabetic mice: the area under the curve for a glucose tolerance test was significantly less (*p* < 0.05) in diabetic mice consuming 300 g raw adzuki bean flour/kg BW than for those that did not consume raw adzuki bean flour. Yet, no significant differences were found between diabetic mice that consumed 300 g cooked adzuki bean flour/kg BW and those that did not consume adzuki bean flour (*p* > 0.05). Thus, food processing, via steaming, seemed to lower the ability of adzuki bean flour to manage T2D outcomes.

#### 4.5.2. Effectiveness of Extrusion Cooking on the Antidiabetic Capacity of Adzuki Bean Supplements

On the other hand, Yao et al. [43] reported that extrusion-cooked adzuki beans (16% moisture content, processed between 80 °C and 150 °C, 160 rpm screw speed) had better antidiabetic benefits than raw adzuki beans. Although the raw adzuki bean had higher total phenolic and total flavonoid contents than cooked extruded adzuki beans, cooked extruded beans had higher polysaccharide and protein contents. Extrusion-cooked adzuki beans also had a higher α-glucosidase inhibitory activity, suggesting a better ability to gradually release glucose into the bloodstream [43]. In fact, both the polysaccharides and proteins from extruded adzuki beans were found to have a higher α-glucosidase inhibitory activity than that from the raw adzuki beans. Both the non-diabetic rats and streptozotocin-induced male diabetic rats that received 200 mg extruded adzuki bean extract/kg BW showed a reduction in postprandial blood glucose as compared to their control counterparts. Compared to rats in the control group, normal rats that received extruded adzuki bean extract had significantly lower (*p* < 0.05) blood glucose levels 30 min after receiving 2 g of sucrose, while there was no significant difference (*p* > 0.05) in blood glucose levels in rats that received raw adzuki bean extract. Similar results were found in the diabetic rats. Compared to diabetic rats in the control group, diabetic rats that received extruded adzuki bean extract had significantly lower (*p* < 0.05) blood glucose levels thirty minutes and one hour after receiving 2 g sucrose, while there was no significant difference (*p* > 0.05) in blood glucose in rats that received raw adzuki bean extract. The results suggested that processing adzuki bean polysaccharides and proteins contributed to the beans’ antidiabetic capacity by increasing the inhibitory activity of α-glucosidase.

#### 4.5.3. Effectiveness of Germination on the Antidiabetic Capacity of Adzuki Bean Supplements

Another way to process adzuki beans is germination, a food processing technique that alters the nutritional and functional properties of the seeds. An animal study conducted by both Jiang et al. [46] and Zhang et al. [47] used germination (31 °C for 48 h, humidity of 95 ± 3%, sprayed with 2 mmol/L monosodium glutamate once every hour) to increase the GABA content in the adzuki beans, and reported that germinated GABA (γ-aminobutyric acid)-rich adzuki beans had an anti-diabetic impact. Jiang et al. [46] found that a high-fat diet significantly increased the serum glucose and HOMA-IR levels while significantly decreasing insulin and HOMA-β levels of the mice (*p* < 0.05). Providing the mice with 35 g adzuki bean/100 g diet for 5 weeks resulted in no statistically significant differences in serum glucose and insulin levels, while significantly lowering the HOMA-IR level of the mice. Providing the mice with 25 g or 35 g GABA-enriched sprouted adzuki beans/100 g diet for 5 weeks significantly lowered the serum glucose and HOMA-IR levels, while significantly increasing the insulin level (*p* < 0.05). Zhang et al. [47] further found that a high-fat diet significantly increased the blood glucose of the fasting mice (*p* < 0.05); however, providing the mice with 35 g GABA-enriched sprouted adzuki beans for 2 and 4 weeks significantly lowered their fasting blood glucose (*p* < 0.05). These results suggested that processing adzuki beans, via germination, contributed to the beans’ antidiabetic capacity by increasing their anti-diabetic polyphenol contents. The impact of food processing on the antidiabetic potential of adzuki bean bioactive compounds is still inconclusive. As a result, studies investigating how various food processing techniques contribute to the alteration of the content of the anti-diabetic adzuki bean bioactive components and their respective antidiabetic potential are still needed.

### 4.6. The Antidiabetic Capacity of Adzuki Bean Supplements, Comparing Their Capacity to Other Means Used to Manage T2D

#### 4.6.1. Effectiveness of Adzuki Bean Hot Water Extract on T2D Outcomes when Compared to Sulfonylurea

Animal studies demonstrated the anti-diabetic capacity of adzuki bean supplementation by comparing its capacity to that of other means used to manage T2D. An animal study explored the impact of hot water extract from adzuki beans on glucose response in non-diabetic mice and streptozotocin-induced diabetic rats, and compared such effects to that of sulfonylurea, an oral antidiabetic medication used in managing T2D [42]. Non-diabetic mice receiving hot water extract (100 mg and 500 mg hot water extract from adzuki beans/kg BW) from adzuki beans had significantly lower postprandial blood glucose than those receiving tolbutamide 30 min after the sucrose administration (*p* < 0.05). Moreover, insulin secretion tended to decrease significantly in mice provided with 500 mg hot water extract from adzuki beans/kg BW. No significant differences (*p* > 0.05) were found in postprandial blood glucose and serum insulin levels in mice receiving hot water extract from adzuki beans and mice receiving the control treatment after the glucose administration. Diabetic rats that received 500 mg hot water extract from adzuki beans/kg BW had significantly lower postprandial blood glucose than diabetic rats that received tolbutamide 30 min and 60 min after the sucrose administration (*p* < 0.05). In addition, no significant difference (*p* > 0.05) was found in postprandial blood glucose levels in diabetic rats receiving 100 mg hot water extract from adzuki beans and rats receiving sulfonylurea treatment after the sucrose administration. Also, no significant difference was found in postprandial blood glucose levels in diabetic rats receiving 100 mg and 500 mg hot water extract from adzuki beans and diabetic rats receiving sulfonylurea treatment after the glucose administration (*p* > 0.05). These results suggested that consuming 500 mg hot water extract from adzuki beans/kg BW might have equivalent or better antidiabetic benefits than consuming sulfonylurea in both healthy and diabetic subjects. 

#### 4.6.2. Effectiveness of Adzuki Bean Polysaccharides on T2D Outcomes when Compared to Metformin

An animal study found that 400 mg/kg BW/day of adzuki bean polysaccharide supplementation could provide similar antidiabetic outcomes as metformin, which is an oral anti-diabetic medication [37]. Providing diabetic rats with 400 mg/kg BW/day of adzuki bean polysaccharides significantly lowered their glucose tolerance, serum insulin level, and insulin resistance when compared to that of the diabetic rats receiving metformin treatment (*p* < 0.05). In addition, diabetes significantly lowered the rats’ hepatic glycogen content (*p* < 0.05). Yet, diabetic rats that received 400 mg/kg BW/day of adzuki bean polysaccharides and diabetic rats that received metformin had a higher hepatic glycogen content than the diabetic rats that did not receive any treatment, and there was no significant difference in the hepatic glycogen content in diabetic rats that were treated with adzuki bean polysaccharides and those treated with metformin (*p* > 0.05). 

#### 4.6.3. Effectiveness of Adzuki Bean Convenience Food on T2D Outcomes when Compared to Traditional Diabetic Low-Glycemic-Index Diets

Like the results demonstrated in the rodents, a randomized, controlled-intervention human trial with 120 T2D patients suggested positive results in the antidiabetic capacity of adzuki bean supplementation in human subjects [29]. In a clinical study, Liu et al. [29] found no significant difference in fasting blood glucose, HbA1C, glycated albumin, fasting insulin, and insulin resistance index of the study participants who consumed the traditional diabetic low-glycemic-index diet and those who consumed an extruded adzuki bean convenience food (*p* > 0.05). Liu et al. [29] provided 44.8 g of adzuki bean extract/day in the form of extruded adzuki bean convenience food (vermicelli, instant powder, and hard candy) to the participants during the four-week intervention period. A study conducted by the same research group reported that 1 g of adzuki bean extract contained 20.99% protein, 159.32 mg of polysaccharides, 0.45 mg of total phenolic contents, and 0.12 mg of total flavonoid [43]. The adzuki bean extract was found to have a 66.48% α-glucosidase inhibitory activity. The traditional diabetic low-glycemic-index diet was used as a means to manage T2D [49]. As shown by the results of Liu et al. [29], the extruded adzuki bean convenience food that provided 44.8 g of adzuki bean extract/day exhibited similar antidiabetic functions as the food with a low glycemic index. In other words, the supplemental consumption of 9.4 g of adzuki bean protein, 7.14 g of adzuki bean polysaccharides, 20.16 mg of adzuki bean total phenolic content, and 5.38 mg of adzuki bean total flavonoid content per day could provide antidiabetic benefits in managing T2D outcomes. 

In sum, results from these studies further suggest the possibility of using adzuki bean supplementation to manage glucose levels in patients with T2D. Although current investigation demonstrates the potential of adzuki beans in managing T2D and glucose metabolism, the underlying pathways related to the absorption and metabolism of sugars are not fully understood. Therefore, more studies are needed to explore how bioactive compounds from adzuki beans contribute to the antidiabetic mechanism.

### 4.7. The Antidiabetic Capacity of Adzuki Bean Supplementation via Improving Liver and Pancreatic Function

#### 4.7.1. Effectiveness of Adzuki Bean Supplementation on Improving Pancreatic Function

Adzuki bean consumption could also improve pancreatic function. Mice that received an adzuki bean extract in their diet were found to have better glucose regulation and pancreatic function [10,38,39]. An animal study providing 3-week-old male C57BL/6 mice with a high-fat diet consisting of 45% kcal from fat found that the high-fat diet significantly increased fasting blood glucose level, serum insulin, insulin resistance, and the Langerhans islet area of β-cells (*p* < 0.05) [38]. Adding 200 mg/kg BW/day of adzuki bean extract to the high-fat diet of the mice significantly reduced the increased parameters (*p* < 0.05). Furthermore, mice that consumed the adzuki bean-supplemented high-fat diet experienced a significant reduction in the SOD1 level (*p* < 0.05), which contributed to lower insulin resistance by reducing oxidative stress. An in vitro study also found that the pancreatic RINm5F cells treated with more than 0.05 mg black adzuki bean ethanolic extract/mL and 25 mM D-glucose secreted significantly more insulin than cells that were treated with 25 mM D-glucose alone (*p* < 0.05), suggesting a better pancreatic β-cell function in the cell population treated with a black adzuki bean ethanolic extract [39]. Moreover, compared to their counterparts that received the high-fat diet, male C57BL/6J mice that received a high-fat diet supplemented with 1 g black adzuki bean ethanolic extract/100 g diet/day for 12 weeks had a significantly less area under the curve in the oral glucose tolerance test (*p* < 0.05), suggesting an improvement on glucose tolerance in mice consuming the extract. Mice receiving a high-fat diet and black adzuki bean ethanolic extract also had significantly lower fasting serum glucose, serum insulin, and HOMA-IR index than mice receiving a high-fat diet (*p* < 0.05). Furthermore, similar antidiabetic benefits were found in mice receiving a high-fat diet supplemented with 0.08 g kaempferol/100 g diet/day, which is an adzuki bean polyphenol. In a histological analysis conducted by Zhao et al. [10], adzuki bean flour supplementation could alleviate the pancreatic damage caused by diabetes. All these results suggest that azuki bean supplementation could enhance pancreatic function and contribute to the prevention and management of T2D.

#### 4.7.2. Effectiveness of Adzuki Bean Supplementation on Improving Liver Function

Adzuki bean consumption could also play a role in improving liver function. Two liver proteins, alanine aminotransferase and aspartate aminotransferase, are used to indicate liver function. Zhao et al. [36] found that the activities of these enzymes, increased by the high-fat diet, were significantly reduced after 12 weeks of adzuki bean supplementation (*p* < 0.05), suggesting an improved liver function. Another study found that consuming 150 g cooked adzuki beans/kg diet with a high-fat diet had similar benefits for liver function [32]. Moreover, providing 200 mg adzuki bean extract/kg BW/day to mice on a high-fat diet significantly reduced the increased serum alanine aminotransferase caused by the high-fat diet (*p* < 0.05) [38]. Lee et al. [38] found that consuming 200 mg/kg BW/day of adzuki bean extract could significantly reduce hepatic inflammation in the mice (*p* < 0.05), and, therefore, improve liver function. Similarly, consuming adzuki bean flour supplements could improve the liver damage caused by diabetes [10]. On the other hand, consuming extruded adzuki bean convenience food seemed to worsen liver function, via inflammation. Liu et al. [29] reported a more severe liver inflammation in T2D subjects after consuming four weeks of extruded adzuki bean convenience food. Because of the contradictory findings of these studies, more research studies on the potential and the extent to which adzuki bean supplements can improve liver function are needed.

### 4.8. The Role of Adzuki Bean Supplements on Insulin Signaling and Glucose Metabolism Pathways

Black adzuki bean ethanolic extract contributed to diabetes management by modulating the glucose metabolic and insulin response of the tissues [39]. Kim et al. [39] found that mice receiving a high-fat diet and black adzuki bean ethanolic extract had a lower gene expression of *Pdk4* than mice receiving only the high-fat diet, supporting the role of adzuki bean ethanolic extract in increasing glucose utilization and glycolysis. On the other hand, the gene expression of *Irs2*, *Irs4*, and *Sort1* was higher in mice receiving a high-fat diet and black adzuki bean extract than in mice receiving only the high-fat diet, suggesting adzuki bean extract may enhance insulin signaling and glucose uptake. 

Adzuki bean polysaccharides could modulate T2D-related outcomes via activation of the PI3K/AKT signaling pathway [37]. Diabetic rats had significantly lower *Insr*, *Irs-1*, *Pi3k*, *Akt*, and *Glut-2* mRNA expression than rats without diabetes (*p* < 0.05), suggesting lower insulin signaling responses. On the other hand, adzuki bean polysaccharides had dose-dependent effects on improving the expression of the genes related to insulin signaling responses in the PI3K/AKT signaling pathway. Male diabetic rats treated with 400 mg/kg body weight/day of adzuki bean polysaccharides had a significantly higher *Insr*, *Irs-1*, *Pi3k*, *Akt*, and *Glut-2* mRNA expression than the diabetic rats, while rats treated with 200 mg adzuki bean polysaccharides/kg BW/day had a significantly higher *Irs-1*, *Pi3k*, *Akt*, and *Glut-2* mRNA expression than the diabetic rats (*p* < 0.05). Rats treated with 100 mg adzuki bean polysaccharides/kg BW/day had significantly higher expression levels of only *Akt* and *Glut-2* (*p* < 0.05). Furthermore, the dose-dependent effects of adzuki bean polysaccharide supplementation were clearer in the expression of insulin-signaling-related proteins in the PI3K/AKT signaling pathway. Male diabetic rats treated with 400 mg/kg BW/day of adzuki bean polysaccharides had a significantly higher INSR, IRS-1, PI3K, P-AKT, and GLUT-2 protein expression than the diabetic rats, while rats treated with 200 mg/kg BW/day of adzuki bean polysaccharides had a significantly higher INSR and P-AKT protein expression than the diabetic rats (*p* < 0.05). Diabetic rats treated with 100 mg/kg BW/day of adzuki bean polysaccharides had a significantly higher level of P-AKT protein expression only when compared to the diabetic rats (*p* < 0.05). Thus, a higher dose of adzuki bean polysaccharides might have a greater impact on improving insulin resistance. 

In addition to managing T2D outcomes via modulating the glucose metabolic process and insulin response of the tissues, adzuki bean bioactive compounds could influence the postprandial glycemic response by regulating the breakdown of complex carbohydrates. Two studies reported the anti-diabetic potential of adzuki bean phenolic extract by inhibiting the activity of α-glucosidases, intestinal enzymes that break down complex carbohydrates into glucose [44,50]. Sreerama et al. [50] found that both black and red adzuki beans inhibited α-glucosidases activities. They reported that the half maximal inhibitory concentration values were 26.28 and 319.22 mg/mL for the black and red adzuki beans, respectively. They also identified anthocyanin as a major contributor to this inhibition, as black adzuki beans had higher anthocyanin contents. Similarly, Yao et al. [44] observed that 10 mg/mL of extruded adzuki bean protein inhibited 60.44% of rat intestinal α-glucosidases (60.44%) in vitro. Liu et al. [48] also examined the effects of adzuki bean flavonoids, saponins, and adzuki bean total extract on T2D outcomes via inhibiting the α-glucosidase activity. The α-glucosidase activity assay showed that adzuki bean flavonoids, saponins, and total extract all had a dose-dependent inhibitory effect on α-glucosidase. Assay results showed that these three components had a significantly higher percentage of α-glucosidase inhibition than the negative control. The related mechanism of action is highlighted in Table 2.

In summary, studies showed that adzuki bean supplementation could help manage T2D. Some studies showed that adzuki bean supplementation had an equivalent or better impact on T2D outcomes than other means of T2D management such as T2D medications and the traditional diabetic low-glycemic-index diets. However, most studies in the current literature were conducted using rodents. Therefore, more human research studies examining the effects of adzuki bean supplementation on T2D-related outcomes are needed.

Because T2D is highly prevalent, its prevention is of equal significance to its management. While current literature suggests the potential use of adzuki bean supplements in the management of T2D, no research has examined their specific role in preventing the onset of the condition. Therefore, investigating the direct influence of adzuki bean supplementation on two common risk factors, obesity and dyslipidemia, for T2D could provide valuable insights into the potential of adzuki bean supplements for preventing the development of T2D.

Table 3 shows available studies (n = 21) on the effect of adzuki bean on obesity and dyslipidemia. Most are rodent studies (n = 18) and there is only one clinical investigation in humans. Two other are in vitro studies. Specific results from these investigations are presented below.

## 5. Role of Adzuki Bean Supplementation in Managing Obesity Outcomes

**Figure 2:**
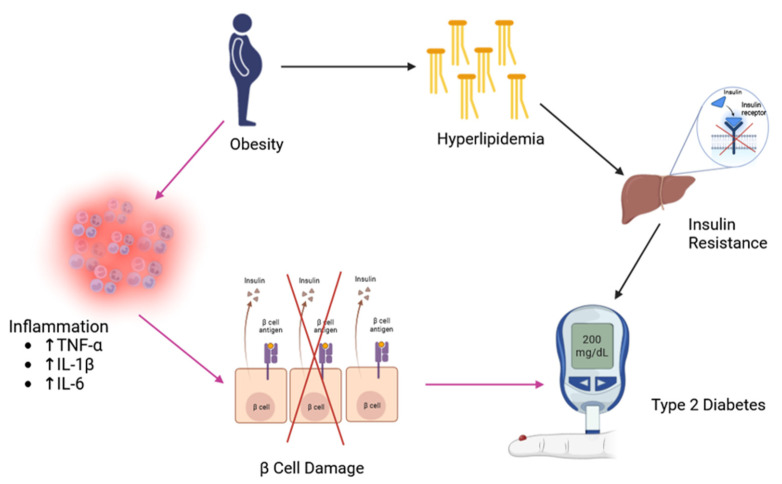
Relationship between obesity and T2D. Information adapted from Saad et al. [58]. “↑” indicates increase.

Weight loss has been demonstrated to be beneficial in preventing and managing diabetes [57]. Indeed, obesity is a well-known risk factor for T2D. Saad et al. [58] highlighted the relationship between obesity and T2D (Figure 2). Obesity levels of individuals can be determined by body mass index (BMI). A study found that the higher the BMI, the higher the prevalence of T2D in older adults in India [59]. Compared to normal-weight individuals, obese individuals had an 8.13-times higher risk of having diabetes. Other publications also highlighted the impact of obesity on the risk of diabetes across various populations [60,61,62,63,64,65]. Thus, weight management is a means to reduce the risk for T2D, and adzuki bean consumption might be a possible means to reduce the risk and prevent T2D via its capacity to manage weight. Studies were conducted to determine whether adzuki bean consumption can manage obesity. Unfortunately, unlike the uniform results observed in studies investigating the association between adzuki bean supplementation and T2D outcomes, the investigations into the relationship between adzuki bean supplementation and its potential anti-obesity benefits have been diverse and inconclusive. The types and the dosage of the adzuki bean supplements provided, as well as the duration of the treatment, seemed to contribute to the weight management capacity of adzuki bean supplements.

### 5.1. Effectiveness of Adzuki Bean Flour on Obesity Outcomes

Adzuki bean flour was used to determine whether it could play a role in managing obesity in animals. While some studies reported that adzuki bean flour consumption had anti-obesity benefits, other studies showed no impact on obesity management [10,32,36,46,47]. Furthermore, the study findings varied, depending on how the adzuki bean supplementation was processed. Zhao et al. [36] found that 12-week consumption of a high-fat diet significantly increased the body weight, the body fat ratio, and the perirenal, epididymal, and retroperitoneal white adipose tissue (WAT) mass in 4-week-old male C57BL/6 mice (*p* < 0.01). However, adding 150 mg adzuki bean supplement/kg diet/day to the high-fat diet significantly decreased body weight (*p* < 0.01), perirenal WAT mass (*p* < 0.01), epididymal WAT mass (*p* < 0.01), retroperitoneal WAT mass (*p* < 0.01), and body fat ratio, which were increased by the high-fat diet (*p* < 0.05). Additionally, Jiang et al. [46] and Zhang et al. [47]) reported that providing mice with a 35 g GABA-enriched sprouted adzuki beans/100 g diet lowered significantly the body weight increased by a high-fat diet (*p* < 0.05). A mouse study that had a 12-week treatment period also found that a high-fat diet significantly increased weight gain, epididymal fat weight, perirenal fat weight, retroperitoneal fat weight, and adipose cell size of the 4-week-old C57BL/6 mice (*p* < 0.01) [32]. After providing the mice with 15% cooked adzuki bean supplementation for 12 weeks, the body weight (*p* < 0.01), the epididymal fat weight (*p* < 0.05), the perirenal fat weight (*p* < 0.05), and the adipose cell size of the mice decreased significantly (*p* < 0.01), but their retroperitoneal fat weight was not different (*p* > 0.05).

Another animal study demonstrated the capacity of cooked adzuki beans to improve weight after short treatment [10]. Diabetic mice were found to have a significantly lower body weight than the healthy nondiabetic mice (*p* < 0.05); yet, after providing the diabetic mice with 300 g cooked adzuki bean flour/kg BW for 8 weeks, their body weights significantly increased (*p* < 0.05). However, providing the diabetic mice with 300 g raw adzuki bean flour/kg BW for 8 weeks did not have any impact on their body weight. The results suggest the importance of the food processing method applied to the adzuki bean flour in relation to its ability to maintain an anti-obesity capacity. These data also indicate that a treatment of short duration with adzuki bean flour does not seems to have anti-obesity capacity.

### 5.2. Effectiveness of Adzuki Bean Extract on Obesity Outcomes

Research studies involving the use of adzuki bean extract on animals also yielded uncertain outcomes regarding the effectiveness of adzuki bean extract in addressing obesity. Some studies reported positive results on the efficacy of adzuki bean supplementation in reducing obesity [38,39,48,52,55]. Lee et al. [38] found that providing mice with 200 mg adzuki bean extract/kg BW/day significantly decreased the fat mass increased by a 12-week consumption of a high-fat diet (*p* < 0.05). A similar study that provided mice with 0.08 g black adzuki bean kaempferol/100 g diet/day or 1 g black adzuki bean extract/100 g diet/day supplemented with the high-fat diet for 12 weeks reported anti-obesity benefits [39]. The researchers found that mice receiving both a high-fat diet and an adzuki bean supplement had a significantly lower weight gain than mice receiving a high-fat diet alone (*p* < 0.05). In another mice study conducted by Kim et al. [55], supplementing a high-fat diet with 1 g black adzuki bean extract/100 g diet/day for 12 weeks significantly reduced the final body weight, the epididymal WAT weight and size, and the mesenteric WAT weight and size, which were increased by the high-fat diet (*p* < 0.05). Likewise, in an animal study that investigated the benefits of adding black adzuki bean extract to a high-fat diet, the researchers reported that a high-fat diet significantly increased the body weight and the epididymal fat weight of 4-week-old male C57BL/6 mice (*p* < 0.05) [52]. However, after providing the mice with 1% black adzuki bean extract/day or 2% black adzuki bean extract/day for 12 weeks, the body weight and the epididymal fat weight of the mice significantly decreased (*p* < 0.05). Moreover, an animal study showed that providing mice with total adzuki bean extract, adzuki bean flavonoids, and adzuki bean saponins along with the high-fat diet for four weeks could significantly reduce their final body weight, parametrial adipose tissue weight, and perirenal adipose tissue weight (*p* < 0.05) [48]. 

The dosage of the adzuki-bean-extract supplement also plays a role in weight management. Two animal studies reported dose-dependent anti-obesity effects of adzuki bean extract [53,54]. Chio et al. [53] found that a high-fat diet significantly increased the final body weight and body weight gain of the 5-week C57BL/6J male mice (*p* < 0.05). After providing the mice with 200 mg adzuki bean supplement for four weeks, their final body weight and body weight gain became significantly lower than that of their control counterparts (*p* < 0.05). However, no statistical differences were found in final body weight and body weight gain between mice that received 100 mg adzuki bean supplement for four weeks and mice in the control group. Another animal study that provided 4-week-old male Sprague Dawley rats with ethanolic extract from black adzuki beans found similar results [54]. A high-fat diet significantly increased the body weight of the rats over 8 weeks (*p* < 0.05). In week two and week three, providing rats with 2% black adzuki bean extract significantly reduced the body weight of the rats that consumed the high-fat diet (*p* < 0.05), while no significant weight reduction was found in rats that consumed 1% black adzuki bean extract along with the high-fat diet (*p* > 0.05). Moreover, a high-fat diet significantly increased the epididymal fat of the rats, while providing rats with 2% black adzuki bean extract significantly reduced the epididymal fat of the rats that consumed the high-fat diet (*p* < 0.05). Yet, the epididymal fat of the rats that consumed 1% black adzuki bean extract and rats that consumed only the high-fat diet was not statistically different. On the other hand, the researchers reported that providing rats with black adzuki bean extract for 8 weeks significantly reduced the body weight of the rats that consumed the high-fat diet (*p* < 0.05), and there was no significant difference in body weight between rats that received 1% black adzuki bean extract and rats that received 2% black adzuki bean extract (*p* > 0.05), suggesting that the length of supplementation also contributed to the ability of the black adzuki beans to manage weight.

In addition to the supplementation dosage, hot water extract from adzuki beans did not have an impact on weight reduction. It is possible that food processing lowers the anti-obesity ability of the adzuki bean extract. Kitano-Okada et al. [31] observed no significant difference in body weight gain between the rats in the control group and rats in the treatment group that received adzuki bean hot water extract for four weeks (*p* > 0.05). Moreover, a higher supplement dosage and a longer supplement duration did not enhance the anti-obesity effect of adzuki bean supplementation. Itoh et al. [41] reported that supplementing hot water extract from adzuki beans into a regular diet for seven weeks had no impact on the mice’s body weight. Furthermore, compared to their control counterparts, mice receiving 500 mg and 5000 mg hot water extract from adzuki beans/kg BW/day had no significant difference in body weight from weeks one to seven (*p* > 0.05).

These data from animal studies indicate that there is no consistency in the effect of adzuki bean extracts on weight gain and obesity.

### 5.3. Effectiveness of Adzuki Bean Polysaccharides and Protein on Obesity Outcomes

Adzuki bean polysaccharides and untreated protein do not seem to have weight management capacity. A 4-week animal study reported no significant difference in body weight in rats treated with low, middle, and high doses of adzuki bean polysaccharides in diabetic rats on days 1, 7, 14, 21, and 28 (*p* > 0.05) [37]. In addition, Yao et al. [44] observed no significant difference in body weight between diabetic mice receiving extruded adzuki bean protein for 6 weeks and diabetic mice that were in the control group (*p* > 0.05). On the other hand, processed adzuki bean protein has the potential to reduce body weight. Zhao et al. [45] provided 4-week-old male C57BL/6 mice with heat-treated adzuki bean protein hydrolysates for 12 weeks to examine the impact of such supplementation on obesity [45]. They found that the weight gain, epididymal fat weight, perirenal fat weight, retroperitoneal fat weight, and mesenteric fat weight significantly increased in mice consuming a high-fat diet (*p* < 0.01); however, providing the adzuki bean protein hydrolysates to mice consuming a high-fat diet significantly lowered their weight gain, epididymal fat weight, perirenal fat weight, retroperitoneal fat weight, and mesenteric fat weight (*p* < 0.01). Also, from weeks 8 to 12, mice that received adzuki bean protein hydrolysates with the high-fat diet had a significantly lower weight than mice that received only the high-fat diet (*p* < 0.05).

Generally, body weight and fat mass are obesity-related outcomes used by research studies to determine the potential of adzuki beans in managing obesity. The dosage of the adzuki bean supplement, the length of the supplementation, and the food processing of the supplements seem to play a role in determining the anti-obesity capacity of the various adzuki bean supplementations. However, with current evidence, the recommended dosage and duration for adzuki bean consumption are still inconclusive. Similarly, studies on the types of adzuki bean supplements that demonstrate anti-obesity capacity were also inconclusive, due to the lack of studies on adzuki bean supplementation. Heat-treated adzuki bean extract seems to have less anti-obesity capacity, while heat-treated adzuki bean protein was found to have a better anti-obesity capacity. Thus, more research is needed to help better understand how various food-processing methods affect the bioactivity and the anti-obesity capacity of adzuki bean compounds.

### 5.4. The Role of Adzuki Bean Supplementation in Adipogenesis and Lipolysis

#### 5.4.1. Effectiveness of Adzuki Bean Supplementation on Adipogenesis

Adipogenesis plays a role in obesity development. It is a process in which adipocyte precursor cells proliferate and differentiate into mature adipocytes [66]. Furthermore, regulating adipogenesis could potentially treat obesity [67]. *Ppar-γ* and *C/ebp-α* promote adipocyte differentiation (which promotes adipogenesis), while inducing *Wnt* inhibits adipogenesis [68,69]. Lee et al. [51] found that supplementing hot water extract from adzuki beans into a high-fat diet for 13 weeks contributed to obesity reduction via modulating adipogenesis. The body weight and adipocyte size of the mice receiving both a high-fat diet and adzuki bean extract were significantly lower than those receiving only high-fat diets (*p* < 0.05). Moreover, Western blot results showed that mice that received both a high-fat diet and adzuki bean extract had significantly lower PPAR-γ, C/EBP-α, and AXIN 1 than mice that received only high-fat diets (*p* < 0.05). The protein expression of WNT10B and DVL2, which was significantly decreased by the high-fat diet, was significantly increased in mice that received both a high-fat diet and adzuki bean extract (*p* < 0.05). By activating the Wnt/β-catenin pathway and inhibiting PPAR-γ and C/EBP-α in perigonadal adipose tissue, adzuki bean extract plays a role in inhibiting adipogenesis, and, therefore, in reducing obesity. In like manner, Kim et al. [55] observed that supplementing the high-fat diet with 1 g black adzuki bean extract/100 g diet/day for 12 weeks significantly downregulated the mRNA expression of the lipogenesis-regulating enzymes and transcription factors in the adipose tissues (*p* < 0.05). Indeed, the mRNA expressions of *Ppar-γ*, *Fas*, *Lpl*, and *Cd36* in mice receiving the black adzuki bean extract were significantly lower (*p* < 0.05) than their control counterparts that consumed only the high-fat diet, suggesting a reduced lipogenesis with consumption of black adzuki bean extract. In addition, a cell culture study reported similar results. Kim et al. [56] observed that the 3T3-L1 mouse preadipocytes treated with 1 mg black adzuki bean extract/mL had a significantly lower *C/ebp-β* mRNA expression than cells that were not treated with black adzuki bean extract at hours 24, 48, and 72 (*p* < 0.05). Moreover, treating the cells with 1 mg black adzuki bean extract/mL significantly lowered the triacylglycerol concentration, when compared to the cells that were not treated with black adzuki bean extract at early, terminal, and full-cell differentiation periods (*p* < 0.05). Together, these studies suggest that adzuki bean extract could potentially manage obesity by inhibiting preadipocyte differentiation during adipogenesis. Likewise, adzuki bean polyphenols also exhibit similar effects via inhibiting triglyceride synthesis. Kitano-Okada et al. [31] reported that treating human adipocytes with 500 µg/mL and 750 µg/mL adzuki bean polyphenols significantly reduced the activity of glycerol phosphate dehydrogenase, a key enzyme in triglyceride synthesis, in the adipocytes (*p* < 0.05). This suggests that the anti-obesity capacity of adzuki bean polyphenols may be due to their ability to lower triglyceride synthesis.

#### 5.4.2. Effectiveness of Adzuki Bean Supplementation on Lipolysis

Lipolysis is responsible for the breakdown of triacylglycerol stored in the cells [70]. Therefore, a higher rate of lipolysis might contribute to weight reduction. In a study with mice, Kim et al. [55] observed that supplementing the high-fat diet with 1 g black adzuki bean extract/100 g diet/day for 12 weeks significantly upregulated mRNA expression of the lipolysis-regulating enzymes and transcription factors in the adipose tissues (*p* < 0.05). Indeed, the mRNA expression of *Atgl*, *Hsl*, *Ppar-α*, *Cpt-1α*, *Mcad*, and *Acox* in mice receiving the 1 g black adzuki bean extract was significantly higher than their control counterparts that received only the high-fat diet (*p* < 0.05), suggesting a higher lipolysis with consumption of black adzuki bean extract. Furthermore, an in vitro study showed that providing fat cells with 1 mg/mL total adzuki bean extract, adzuki bean flavonoids, and adzuki bean saponins significantly increased lipolysis (*p* < 0.05) [48]. Although adzuki bean supplementation could increase lipolysis and contribute to weight management, which could potentially reduce the risk of T2D, an increase in basal lipolysis rates could play a role in insulin resistance [71]. Therefore, it is important to be aware of the consumption of adzuki-bean-extract supplement as a preventive measure for T2D, and more research is needed to better understand how adzuki bean extract impacts the lipolysis-related pathway and T2D-related pathway.

## 6. The Role of Adzuki Bean Supplementation on Dyslipidemia Outcomes

According to Franz et al. [72], one of the goals of managing T2D is to improve the lipid profile. Dyslipidemia is a lipid disorder that involves abnormal serum cholesterol and serum triglyceride levels [73]. This disorder is characterized by increased levels of low-density lipoprotein cholesterol (LDL), very low-density lipoprotein cholesterol (VLDL), and triglyceride or triacylglycerol (TG) in the serum [74]. On the other hand, high-density lipoprotein cholesterol (HDL) in the serum is reduced. Dyslipidemia is also one of the conditions experienced by subjects with T2D [36]. Indeed, it is one of the major risk factors for macrovascular complications, such as cardiovascular disease, in patients with T2D [75,76,77]. Moreover, dyslipidemia was found to play a role in the development of T2D. Dyslipidemia has been considered a modifiable T2D risk factor [78], and abnormal lipid levels are indicators of the risk of T2D [79,80]. A human study found that the percentage of T2D subjects who had high total cholesterol (TC), high TG, and low HDL was significantly higher than T2D subjects who had normal TC, TG, and HDL [79]. The odds ratios for these lipids and T2D were also significant. A meta-analysis that pooled data from 15 human studies found that high BMI, TG, TC, LDL, and low HDL were associated with a higher risk of T2D [80]. Therefore, managing abnormal serum-lipid levels not only lowers the risk of T2D, but also improves the quality of life in T2D patients. 

### 6.1. Effectiveness of Adzuki Bean Flour on Dyslipidemia Outcomes

The duration of treatment and the food processing method seem to play roles in the effectiveness of adzuki bean flour supplements in managing various serum lipid levels [10,32,36]. Zhao et al. [36] found that consuming a high-fat diet for 12 weeks increased significantly the serum concentrations of TG, LDL, HDL, and TC (*p* < 0.01). Adding 15% adzuki bean supplement/day to the high-fat diet decreased the serum concentrations of TG (*p* < 0.01), LDL (*p* < 0.05), and TC (*p* < 0.05) which were increased by the high-fat diet. However, no significant difference in blood HDL concentration was found in mice receiving both 15% adzuki bean supplement/day and the high-fat diet and mice receiving a high-fat diet alone (*p* > 0.05). On the other hand, another animal study found that the serum levels of TC, TG, HDL, and LDL were significantly increased by the consumption of a high-fat diet (*p* < 0.01) [32]. Consuming 150 g of cooked adzuki bean for 12 weeks reduced only serum TG (*p* < 0.01). Yet, no significant differences were found in serum TC, HDL, and LDL (*p* > 0.05).

Raw adzuki bean flour seems to have a better capacity to modulate serum lipid levels than cooked adzuki bean flour [10]. Zhao et al. [10] investigated the impact of adzuki bean flour on the serum lipid profile using streptozotocin-induced diabetic mice. Diabetic mice had significantly higher serum TC, LDL, and HDL than nondiabetic mice. For diabetic mice that received 300 g raw adzuki bean flour/kg BW/day for eight weeks, only serum TC and LDL significantly decreased (*p* < 0.05). Moreover, diabetic mice receiving 300 g cooked adzuki bean flour/kg BW/day had a significant decrease only in serum TC. Interestingly, Zhao et al. [10] reported that consuming 300 g/kg BW/day of raw adzuki bean flour supplementation increased serum TG levels in diabetic mice, which contradicted other results (*p* < 0.05). Because of the potential for adzuki bean flour to aggravate the serum TG levels, it is important to be aware of the possible adverse effects of supplementing the diet with adzuki bean flour.

### 6.2. Effectiveness of Adzuki Bean Extract on Dyslipidemia Outcomes

Adzuki bean extract also plays a role in ameliorating dyslipidemia [31,48,55]. Kim et al. [55] observed that a high-fat diet significantly increased the TG, TC, LDL, and VLDL levels in serum (*p* < 0.05); however, supplementing the high-fat diet with 1 g black adzuki bean extract/100 g/day for 12 weeks reduced the serum TG, TC, LDL, and VLDL levels (*p* < 0.05). Kitano-Okada et al. [31] found that compared to their control counterparts, rats receiving 1% adzuki bean extract and the normal diet had significantly lower serum TC, TG, and non-HDL levels after four weeks of adzuki bean treatment (*p* < 0.05). Moreover, rats receiving 1% adzuki bean extract and the high-fat diet had a significantly lower serum non-HDL level after 4 weeks of treatment when compared to their control counterparts (*p* < 0.05). The in vitro study conducted by Kitano-Okada et al. [31] showed that treating the human adipocytes with 250 µg/mL, 500 µg/mL, or 750 µg/mL adzuki bean polymerized polyphenols or unpolymerised polyphenols significantly lowered the TG concentration in the adipocytes (*p* < 0.05). Yet, only cells that were treated with adzuki bean unpolymerised polyphenols showed a dose-dependent effect. Similarly, an animal study showed that providing total adzuki bean extract, adzuki bean flavonoids, and adzuki bean saponins to mice receiving the high-fat diet could reduce their serum TG and TC significantly (*p* < 0.05) [48]. In addition, such supplementation could significantly increase the serum HDL of the mice receiving the high-fat diet (*p* < 0.05). Moreover, compared to mice receiving the high-fat diet, mice receiving 60 mg total adzuki bean extract/kg BW/day, 300 mg total adzuki bean extract/kg BW/day, 300 mg adzuki bean flavonoids/kg BW/day, or 300 mg adzuki bean saponins/kg BW/day had a significantly lower serum LDL.

On the other hand, unlike its impact on T2D outcomes, adzuki-bean-extract supplementation does not seem to contribute to the efficacy of modulating serum lipid levels. Itoh et al. [41] reported that supplementing hot water extract from adzuki beans into a regular diet for seven weeks had no impact on the serum lipid profile of the mice. Compared to their control counterparts, mice receiving 500 mg or 5000 mg hot water extract from adzuki beans/kg BW/day had no significant difference in serum TC, TG, and phospholipid at weeks one, four, and seven (*p* > 0.05). Likewise, another animal study that provided four-week-old male Sprague Dawley rats with ethanolic extracts from black adzuki beans found that a high-fat diet significantly increased the serum TG, TC, HDL, and non-HDL of the rats (*p* < 0.05) [54]. Providing a 1% black adzuki bean extract to rats significantly reduced serum TG, TC, and HDL levels, but not HDL levels when they were on a high-fat diet (*p* < 0.05). Similarly, supplying rats with a 2% black adzuki bean extract significantly decreased their serum TG and non-HDL levels under the same dietary conditions (*p* < 0.05). Although both 1% and 2% doses of black adzuki bean extract had some antidiabetic effect, a higher adzuki bean supplement dosage was found to affect lower serum-lipid levels. These study findings contradicted the presumption that a higher dose of adzuki bean supplementation should have a stronger impact on the outcomes of interest.

### 6.3. Effectiveness of Adzuki Bean Polysaccharides on Dyslipidemia Outcomes

Consuming adzuki bean polysaccharides could potentially manage some of the serum lipid levels [37,40]. Wu et al. [37] reported that rats treated with low and medium adzuki bean polysaccharide doses had a significantly higher serum HDL than diabetic rats on day 28 (*p* < 0.05). Moreover, rats treated with a high adzuki bean polysaccharide dose had a significantly lower serum TG and significantly higher HDL than diabetic rats on day 28 (*p* < 0.05). However, no significant differences in TC and LDL were found in rats treated with adzuki bean polysaccharides and diabetic rats on day 28 (*p* > 0.05). Another study conducted by Wu et al. [40] found that diabetic mice had a significantly higher serum TG level than healthy nondiabetic mice (*p* < 0.01), and that providing the diabetic mice with adzuki bean polysaccharides for four weeks significantly decreased the serum TG level (*p* < 0.01). On the other hand, although diabetic mice had significantly higher serum TC and LDL levels than healthy nondiabetic mice (TC: *p* < 0.05; LDL: *p* < 0.01), there was no significant decrease in serum TC and LDL levels after providing diabetic mice with adzuki bean polysaccharides for four weeks (*p* > 0.05).

### 6.4. Effectiveness of Adzuki Bean Protein on Dyslipidemia Outcomes

Like other functional compounds in adzuki beans, the impacts of adzuki bean protein on serum lipid levels are uncertain. Although Yao et al. [44] found that serum TC and LDL in diabetic mice receiving extruded adzuki bean protein and in diabetic mice in the control group were not significantly different (*p* > 0.05), they found that diabetic mice receiving extruded adzuki bean protein had a significantly lower serum TG and a significantly higher serum HDL than diabetic mice in the control group (*p* < 0.05). Also, the effect of the extruded adzuki bean protein was found to be dose-dependent. Furthermore, Zhao et al. [45] found that mice that received heat-treated adzuki bean protein hydrolysates with the high-fat diet for 12 weeks had significantly lower serum TG, TC, and LDL than their control counterparts that received only the high-fat diet (TG: *p* < 0.01; TC: *p* < 0.05; LDL: *p* < 0.01). However, no significant difference was found in the serum HDL between the two groups (*p* > 0.05).

In general, research indicates that the consumption of different compounds derived from adzuki beans has shown diverse effects on serum lipid levels. These effects vary, based on factors such as the specific type of adzuki bean compound used, the dosage and duration of supplementation, and the methods employed in processing the supplement. As a result, the influence of adzuki bean supplements on individual serum lipid levels is subject to variability. Some studies showed the benefits of adzuki bean supplementation in managing serum lipid levels, while other studies found no benefits in modulating serum lipid levels [10,32,36,41,44]; on the contrary, one study found that consumption of adzuki bean supplements might worsen the serum TG level [10]. Therefore, more research studies are needed to determine whether adzuki bean supplementation could modulate serum lipid levels and alleviate dyslipidemia.

## 7. Effect of Adzuki Bean Supplementation on Gut Microbiota and Oxidative Stress

The gut microbiota and oxidative stress have been demonstrated to be associated with T2D. The severity of gut microbiota dysbiosis and T2D are associated, as the former plays a role in insulin resistance and incretin secretion [81]. Thus, re-establishing gut microbiota harmony could improve T2D symptoms and progression [81]. Disrupting the pro-oxidant-antioxidant system leads to an increase in oxidative stress. Oxidative stress is closely related to the development of T2D via various mechanisms. Oxidative stress plays a role in insulin signal inhibition and adipocytokine dysregulation, and leads to insulin resistance [82]. In addition, oxidative stress in pancreatic β-cells can lead to β-cells apoptosis and β-cells dysfunction. Therefore, reducing oxidative stress could potentially prevent and manage T2D [82]. Therefore, food products with the capacity to modulate the homeostasis of the gut microbiota and the oxidative stress level could be an alternative way to manage and lower the risk of T2D. 

### 7.1. Effectiveness of Adzuki Bean Supplementation on Gut Microbiota Outcomes

Adzuki bean supplementation contributed to the gut microbiota composition [32,36,45,47,51]. A 12-week mouse study reported that a high-fat diet significantly disrupted the gut microbiota composition (*p* < 0.05), and such disruptions in the gut microbiota were alleviated after providing the mice with 15% cooked adzuki bean supplementation for 12 weeks [32]. Other studies reported similar results. Lee et al. [51] found that supplementing 5% adzuki bean hot water extract (whole beans, 90–100 °C, 20 min) into a high-fat diet for 13 weeks contributed to population changes in gut microbes, increasing the abundance of *Lactobacillus* and decreasing the abundance of *Blautia* and *Ruminococcus*. Moreover, Zhao et al. [45] reported that providing heat-treated adzuki bean protein hydrolysates for 12 weeks to the mice consuming a high-fat diet reversed the changes in the gut microbiota population and significantly increased the concentration of beneficial microbial-producing short-chain fatty acids (*p* < 0.05). Likewise, Zhao et al. [36] found that providing 4-week-old male C57BL/6 mice with a high-fat diet (60% kcal from fat) significantly reduced the total number of microbial species in the mice (*p* < 0.05). However, supplementing 150 mg adzuki bean flour/kg diet/day into the high-fat diet for 12 weeks significantly increased the total number of microbial species (*p* < 0.01) [36]. In particular, the abundance of *Bifidobacterium* was enriched significantly by adzuki bean supplementation. An animal study reported that adzuki bean supplementation (a high dietary intake of gamma-aminobutyric acid (GABA)-rich sprouted adzuki bean) caused changes in the abundance of the Firmicutes, Bacteroidetes, Verrucomicrobia, and Akkermansia, species that exhibited the potential to improve T2D outcomes [47], compared to their counterparts that were fed a high-fat diet (*p* < 0.05). These results suggested that supplementation of GABA-rich sprouted adzuki beans could shift the gut microbiota of the T2D mice toward the prevalence of species that could regulate glycolipid metabolism.

Current studies reported that adzuki bean supplementation played a role in modulating the gut microbiota composition, yet, which species responded to the supplementation and how the supplementation influenced the abundance of the affected species is still not clear. Also, there is a lack of research studies investigating changes in the gut microbiota during T2D and the role adzuki bean supplementation plays in modulating these changes. Thus, more research studies that identify the impact of adzuki bean supplementation on the gut microbiota and examine the underlying mechanisms of this impact in a T2D environment are needed. 

### 7.2. Effectiveness of Adzuki Bean on Antioxidative Outcomes

Wang et al. [83] compared the antioxidative capacity of four different foods, including dry adzuki bean, fresh raspberry, dry soybean, and fresh broccoli, using biochemical assays. They found that dry adzuki bean had the lowest EC_50_ value for DPPH and the highest oxygen radical absorbance capacity among all four foods. Moreover, the ferric-reducing antioxidant power (FRAP) of the adzuki bean was the second highest among all four foods. These results suggested that the adzuki bean had high antioxidant capacities, among the foods examined. Moreover, the DPPH scavenging activity and FRAP values of defatted adzuki bean flour were significantly higher (*p* < 0.05) than in the mung bean (adzuki bean: DPPH scavenging activity μmol Trolox equiv/g: 4.44 ± 0.12 vs. mung bean: 3.22 ± 0.11; adzuki bean: FRAP value mmol Fe^2+^ equiv/100 g: 27.39 ± 1.36 vs. mung bean: 23.97 ± 2.36 [50]). Another study reported that the adzuki bean hot water extract had an IC_50_ value of 2.1 µg/mL for DPPH, which was lower than that for vitamin C and vitamin E [41].

Although results from the antioxidative biochemical assays show that adzuki beans exhibit antioxidative capacity, there is a lack of evidence in vivo that supports the antioxidative benefits of consuming adzuki bean supplements. Therefore, more research studies are needed to examine the impacts adzuki bean supplementation has in modulating oxidative stress in cell cultures, animals, and humans.

## 8. Conclusions

The adzuki bean has been employed empirically in some world regions as an oriental medicine for treating various diseases. However, its potential in treating or moderating T2D remains uncertain. The primary objective of this review was to explain the pivotal aspects that contribute to the potential of adzuki bean supplementation in preventing and managing T2D. In general, this review found that consuming adzuki bean supplements could be beneficial in managing T2D outcomes. Yet, this conclusion is based primarily on rodent studies; therefore, controlled clinical trials in humans are needed to confirm the benefits of adzuki bean consumption in T2D patients. 

Several research gaps were identified: (1) supporting data are mostly from animal studies and there is only one investigation in humans; (2) the optimal dosage and duration of treatment for adzuki bean supplementation are inconclusive in the current literature. The existing human study concluded that supplementing 44.8 g of adzuki beans extract/day to the diet exhibited similar antidiabetic functions to food with low glycemic index; however, more clinical human investigations are needed to confirm this finding; (3) the rodent studies exclusively involved male mice, with no inclusion of female animals. As a result, the similarity of the anti-diabetic effects of adzuki bean supplementation in both males and females remains uncertain; and (4) there is a lack of studies examining the underlying mechanisms of the functional compounds within adzuki beans and their effects on pathways associated with T2D. Understanding these mechanisms is crucial for comprehending the potential of adzuki bean supplementation in the management and prevention of T2D. 

Considering the gaps and uncertainties in the current literature, it is important to understand the underlying mechanisms of the adzuki bean compounds, particularly their impacts on T2D-related outcomes. It is essential to determine the viability of adzuki bean supplementation as a potential tool for preventing and managing T2D in both males and females. There is also a need for further research focusing on the chemical and biochemical characterization of functional compounds. Adzuki bean supplementation to prevent the development of T2D is inconclusive, as are its effects in managing obesity and dyslipidemia-related outcomes. Therefore, more human studies are needed, to investigate such topics.

## Figures and Tables

**Table 1:** Examples of commercially available food products that contain adzuki beans.

Country	Name	Description	Image	Ingredients
Japan	Steamed Adzuki Snack-Sweet Red Bean	Steamed adzuki bean	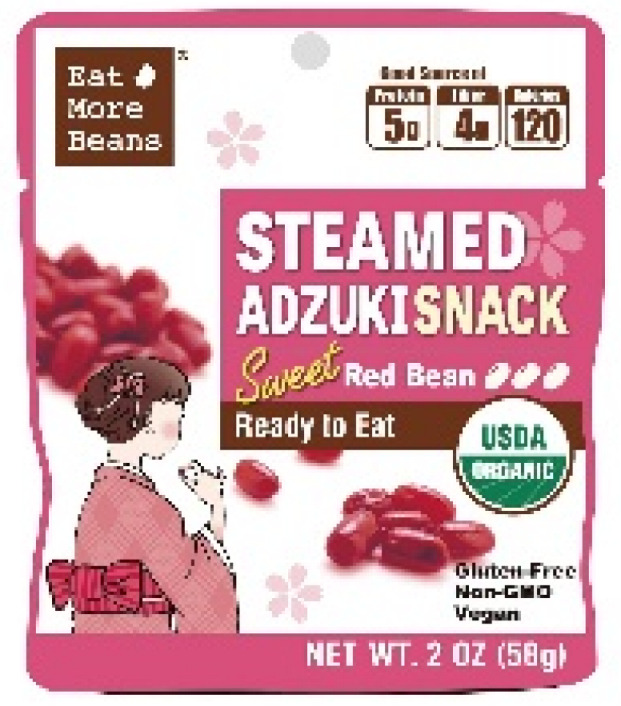	Organic adzuki beans, organic sugar, and water
Taiwan	Taitan Adzuki Bean with Black Glutinous Rice and Quinoa Instant Cereals	Adzuki bean and black glutinous rice mix	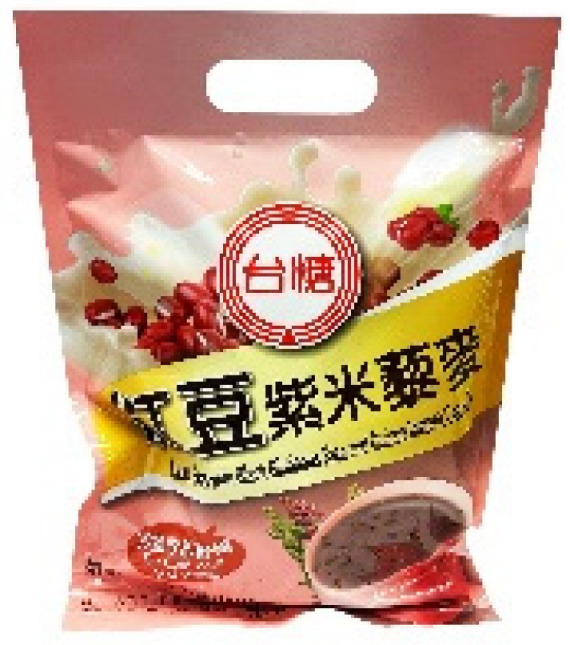	Adzuki bean, milk powder, black glutinous rice, quinoa, and other ingredients
Taiwan	Formosa Yay Imperial Mochi (Red Bean Filling)	Mochi with adzuki bean paste	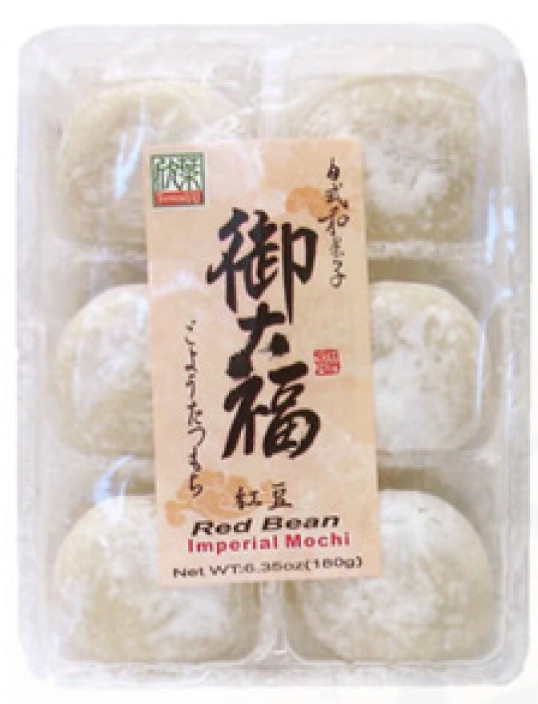	Rice and adzuki bean paste
Japan	Pure Azuki Bean Tea	Adzuki bean tea bag	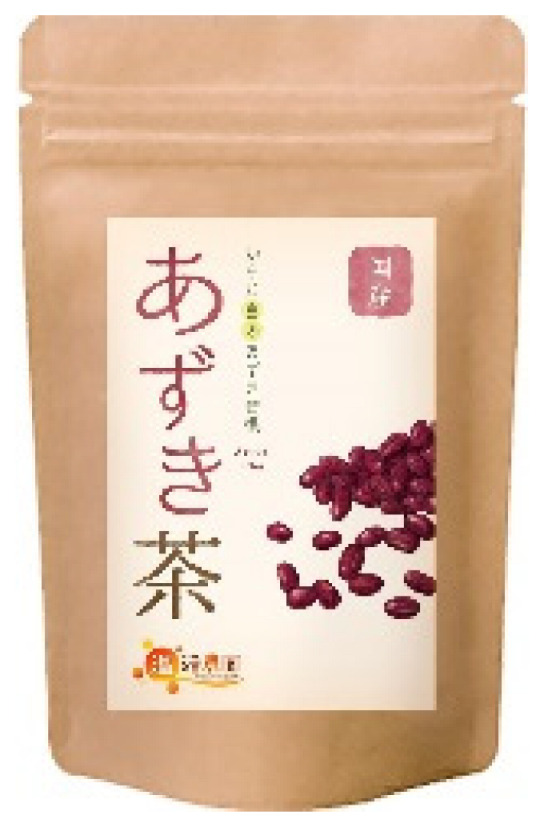	Adzuki bean
Japan	Tenzan Honpo Yokan Tsubuan	Japanese adzuki bean pastry	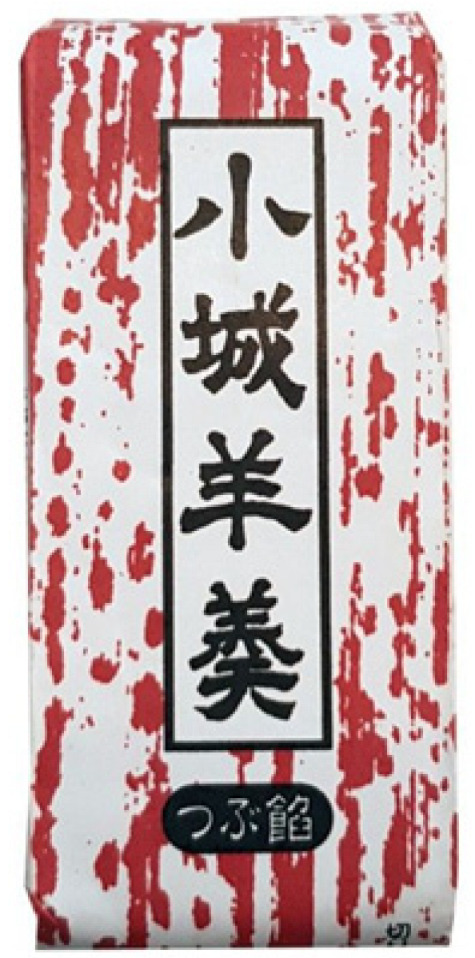	Adzuki bean
Japan	Ogura Komachi	Bread with adzuki bean jam added	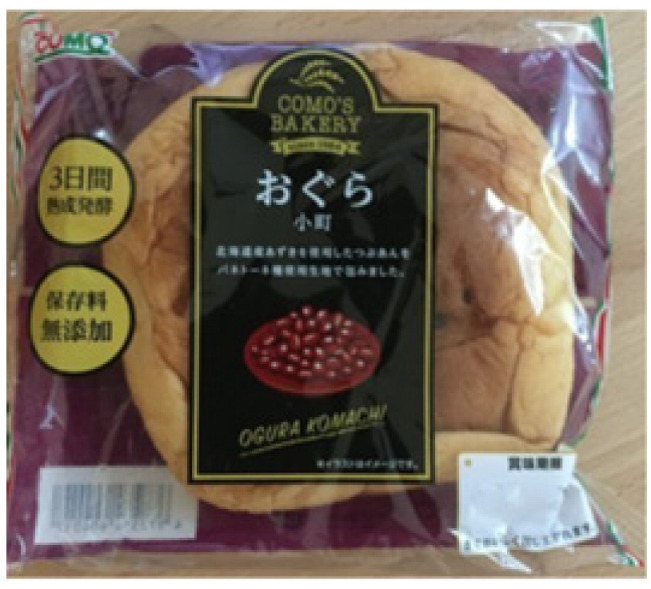	No details provided
Japan	Tohato All Azuki (Red Bean) and Matcha (Green Tea) Snack	Pastry with adzuki bean and matcha added	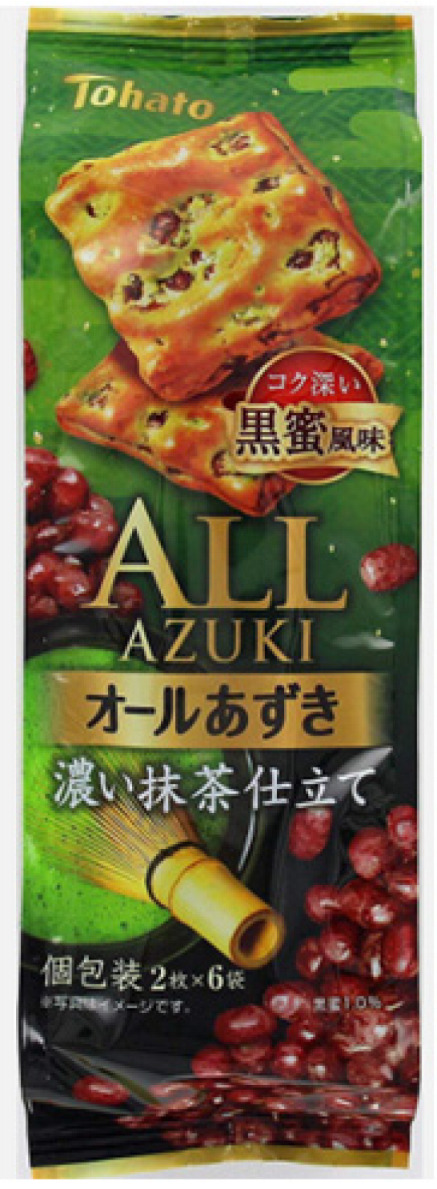	No details provided
Japan	Morokoshi	Molded cake made from adzuki bean flour	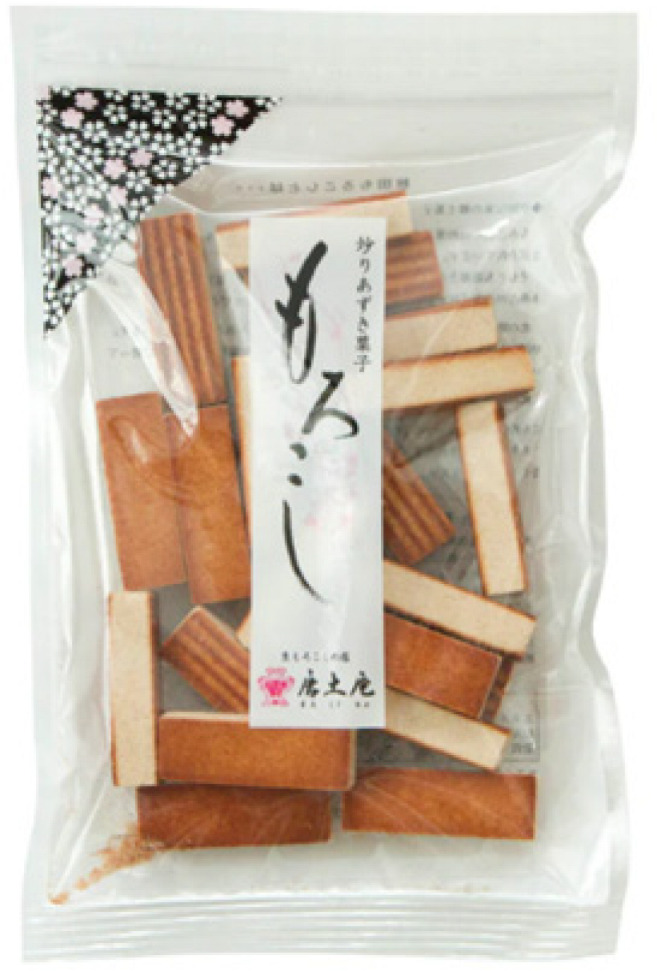	Caster sugar, adzuki bean powder, and white bean paste
Japan	Oshiruko	Freeze-dried sweets adzuki (red bean) soup	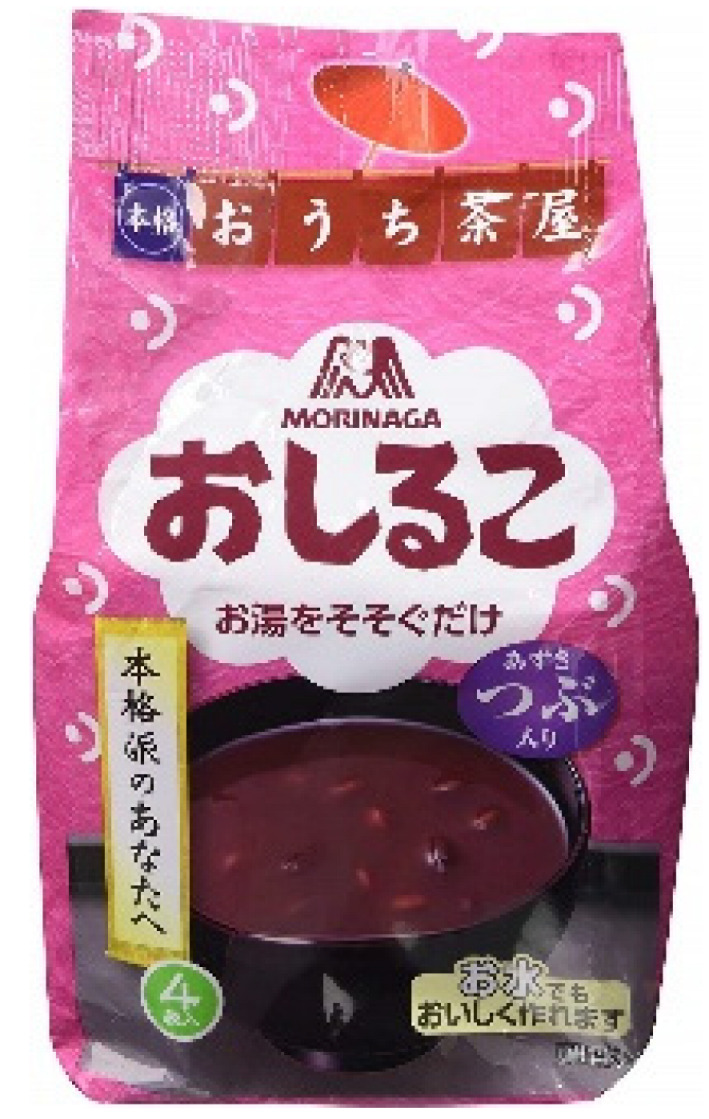	No details provided
Japan	Dorayaki	Japanese pancake with adzuki bean paste filling	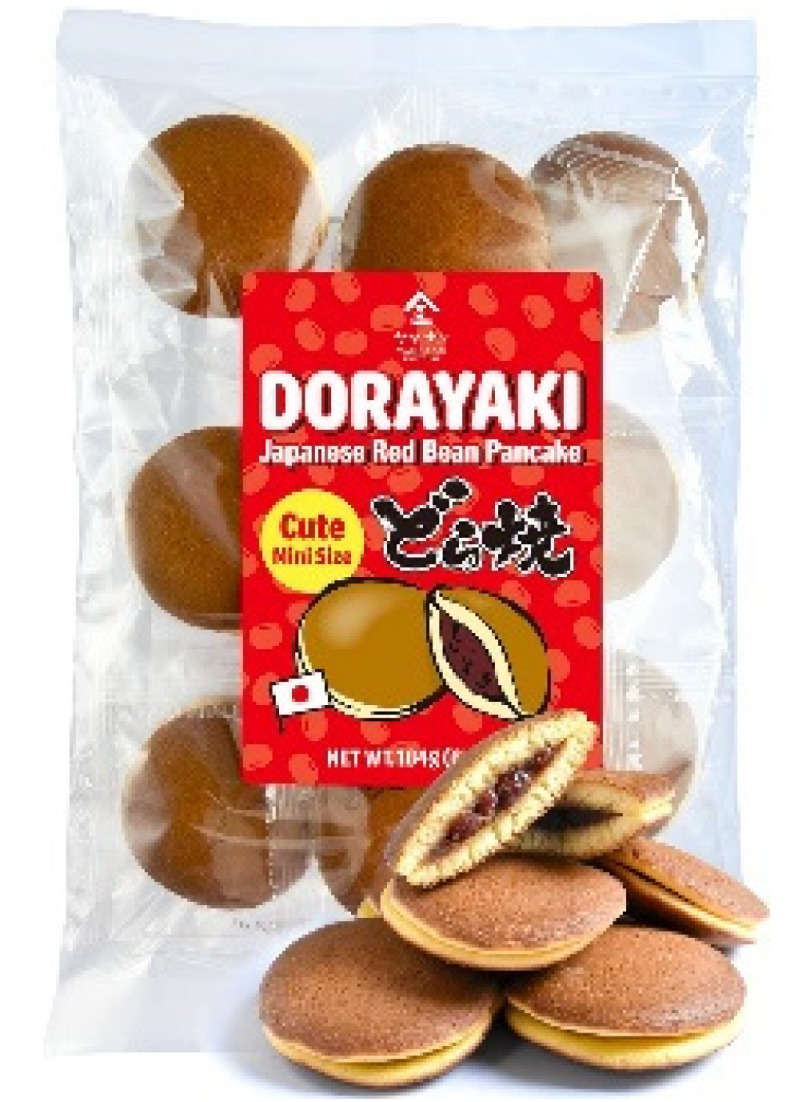	Sugar, egg, wheat, trehalose, adzuki beans, water, sorbitol, reduced sugar syrup, rice flour, glutinous rice flour, modified food starch, vegetable oil, baking powder, and honey
Japan	UHA High Concentrated Milk 8.2 Azuki Milk	Cow milk with adzuki bean flavor added	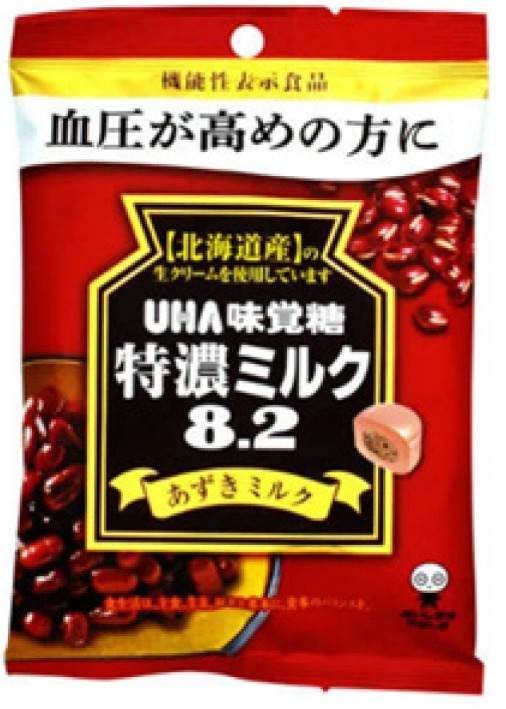	No details provided
Japan	Yamasan Kyoto Uji Japanese Mochi Candy	Candy with adzuki bean flavor added	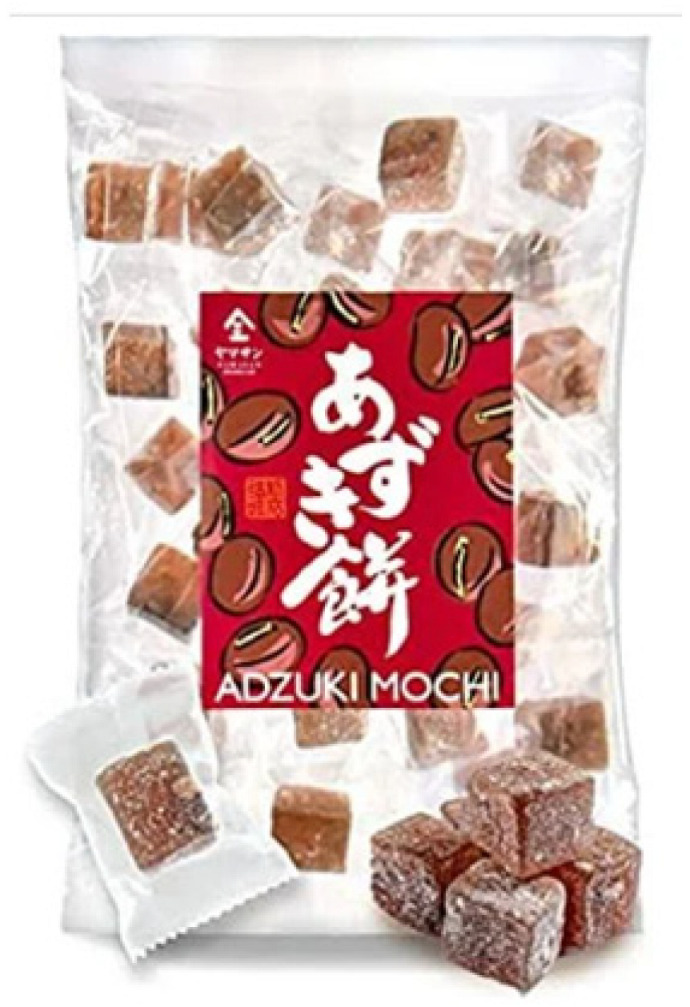	No details provided

**Table 2:** Effects of different adzuki bean supplements on type 2 diabetes outcomes in humans, animals, and in vitro.

Treatment		Outcomes Related to Diabetes or Glucose Metabolism/Pathways			Reference
Dose	Duration	Glucose-related	Insulin-related	Liver/pancreatic function-related and potential mechanism of action	
**Animal studies**					
150 g adzuki bean flour/kg diet/day	12 weeks	↓* fasting blood glucose levels ↓* blood glucose levels during the oral glucose tolerance test	↓* fasting serum insulin levels ↓* HOMA-IR index	The mechanism of action affected: insulin secretion pathway	(Zhao, Hou, Fu, et al., 2021) [36]
200 mg adzuki bean extract/kg BW/day	12 weeks	↓* fasting blood glucose levels	↓* serum insulin levels ↓* insulin resistance	↓* the Langerhans islet area of β cells The mechanism of action affected: insulin signaling pathway	(Lee et al., 2022) [38]
100 mg ABP/kg BW/day 200 mg ABP/kg BW/day 400 mg ABP/kg body BW/day	4 weeks	Medium and high doses of ABP: ↓* fasting blood glucose on days 14, 21, and 28 Low dose of ABP: ↓* fasting blood glucose on day 28 High dose of ABP: ↓* area under the curve for the oral glucose tolerance test ↑* *Glut-2* Medium dose of ABP: ↑* *Glut-2* Low dose of ABP: ↑* *Glut-2*	↓* fasting serum insulin levels ↓* HOMA-IR results High ABP dose only: ↑* *Insr* ↑* *Irs-1* ↑* *Pi3k* ↑* *Akt* Medium dose of ABP only: ↑* *Irs-1* ↑* *Pi3k* ↑* *Akt* Low ABP dose only: ↑* *Akt*	↑* hepatic glycogen content The mechanism of action affected: glucose uptake pathway, and insulin signaling pathway	(Wu et al., 2020) [37]
1 g BAB extract and 0 g kaempferol/100 g diet/day 0 g BAB extract and 0.08 g kaempferol/100 g diet/day	12 weeks	↓* area under the curve for the oral glucose tolerance test ↓* fasting serum glucose	↓* serum insulin ↓* HOMA-IR index - *Pdk4* - *Chi3l3* - *Ppp1r3b* - *Irs2* - *Irs4* - *Sort1* - *Foxp1*	The mechanism of action affected: insulin signaling pathway	(Kim et al., 2016) [39]
400 mg ABP/kg BW/day	4 weeks	3- and 4-week supplementation: ↓* fasting blood glucose levels	↑* insulin sensitivity index	↑* hepatic glycogen - islet cell numbers The mechanism of action affected: gluconeogenesis pathway, and glycogenesis pathway	(Wu et al., 2019) [40]
300 g raw adzuki bean flour/kg BW/day 300 g cooked adzuki bean flour/kg BW/day	8 weeks	300 g/kg raw adzuki bean flour: ↓* fasting blood glucose levels ↓* glycated serum protein levels ↓* the area under the curve for the oral glucose tolerance test		The mechanism of action affected: insulin secretion pathway and glucose uptake pathway	(Zhao, Hou, Laraib, et al., 2021) [10]
500 mg of cellulose/kg BW/day 5000 mg of cellulose/kg BW/day 500 mg of EtEx. 40/kg BW/day 5000 mg of EtEx.40/kg BW/day	7 weeks 4 weeks 1 week	500 mg EtEx. 40 only: ↓* blood glucose levels at weeks 2, 3, 5, and 6 5000 mg EtEx. 40 only: ↓* blood glucose levels from week 1 to 7 5000 mg EtEx. 40 vs. 5000 mg cellulose: - HbA 1c	5000 mg EtEx. 40 vs. 5000 mg cellulose: ↓* plasma insulin concentration	The mechanism of action affected: insulin secretion pathway	(Itoh et al., 2009) [41]
100 mg EtEx.40/kg BW/day 500 mg EtEx.40/kg BW/day	120 min	↓* postprandial blood glucose in healthy mice 30 min after the sucrose administration - postprandial blood glucose in healthy mice after the glucose administration - postprandial blood glucose levels in diabetic rats after the glucose administration 500 mg EtEx.40 ↓* postprandial blood glucose in diabetic mice 30 min and 60 min after the sucrose administration 100 mg EtEx 40 - blood glucose levels in diabetic rats after the sucrose administration	- serum insulin levels in healthy mice after the glucose administration 500 mg EtEx. 40 only: ↓* insulin secretion in healthy mice	The mechanism of action affected: starch digestion and insulin secretion pathway	(Itoh et al., 2004) [42]
200 mg ABE/kg BW/day 200 mg EABE/kg BW/day	120 min	↓* blood glucose levels in healthy rats 30 min after receiving 2g sucrose ↓* blood glucose levels in diabetic rats 30 min and 1 h after receiving 2 g sucrose		The mechanism of action affected: starch digestion	(Yao, Ren, et al., 2014) [43]
1% of EA 2% of EA	6 weeks	↓* blood glucose in diabetic mice ↓* blood glucose in diabetic mice 30 min after 2 g/kg glucose administration		The mechanism of action affected: starch digestion	(Yao et al., 2014) [44]
6 g AP/100 g diet/day	12 weeks	↓* fasting blood glucose ↓* the area under the curve for the oral glucose tolerance test	↓* fasting serum insulin ↓* HOMA-IR index	The mechanism of action affected: starch digestion and insulin secretion pathway	(Zhao et al., 2022) [45]
15 g GABA/100 g diet/day 25 g GABA/100 g diet/day 35 g GABA/100 g diet/day 35 g Adzuki bean/100 g diet/day 0.1 g GABA/kg diet/day 0.1 g Metformin/kg diet/day	6 weeks	↓ fasting blood glucose ↓* serum glucose	↓* HOMA-IR ↑* the insulin level (*p* < 0.05).	The mechanism of action affected: glycine, serine, and threonine metabolism pathway, and ether lipid metabolism pathway	(Jiang et al., 2021) [46]
		↓* fasting blood glucose			(Zhang et al., 2022) [47]
**Human study**					
44.8 g ABE/day	4 weeks	- fasting blood glucose - HbA1C - glycated albumin	- fasting insulin - insulin resistance index	The mechanism of action affected: insulin signaling pathway	(Liu et al., 2018) [29]
**In vitro studies**					
0.025 mg/mL BAB ethanolic extract 0.05 mg/mL BAB ethanolic extract 0.1 mg/mL BAB ethanolic extract 0.2 mg/mL BAB ethanolic extract	96 h		↑* insulin secretion	The mechanism of action affected: insulin secretion pathway	(Kim et al., 2016) [39]
0.25 mg/mL ABTE, ABF, or ABS 0.50 mg/mL ABTE, ABF, or ABS 0.75 mg/mL ABTE, ABF, or ABS 1 mg/mL ABTE, ABF, or ABS	N/A	↑* percentage of α-glucosidase inhibition in ABTE, ABF, and ABS		The mechanism of action affected: starch digestion	(Liu et al., 2017) [48]

↓ decrease/lower/less; ↑ increase/higher/more; - no change; * *p* < 0.05. Polysaccharides from adzuki beans (ABP); black adzuki bean (BAB); hot water extracts from adzuki beans (EtEx. 40); adzuki bean extract (ABE); extruded adzuki bean extract (EABE); extruded adzuki bean protein (EA); heat-treated adzuki bean protein hydrolysates (AP); GABA-enriched adzuki bean (GABA); flavonoids of adzuki bean (ABF); saponins of adzuki bean (ABS); total extract of adzuki bean (ABTE); body weight (BW).

**Table 3:** Effects of different adzuki bean supplements on obesity and dyslipidemia outcomes in animals and in vitro.

Treatment		Outcomes Related to Obesity and Dyslipidemia		Reference
Dose	Duration	Outcomes related to obesity	Outcomes related to dyslipidemia	
Animal studies				
150 g adzuki bean flour/kg diet/day	12 weeks	↓* body weight ↓* perirenal, epididymal, and retroperitoneal white adipose tissue mass ↓* body fat ratio	↓* serum TG, LDL, and serum TC - serum HDL	(Zhao, Hou, Fu, et al., 2021) [36]
200 mg adzuki bean extract/kg BW/day	12 weeks	↓* fat mass		(Lee et al., 2022) [38]
100 mg ABP/kg BW/day 200 mg ABP/kg BW/day 400 mg ABP/kg BW/day	4 weeks	- body weight on days 1, 7, 14, 21, and 28	Low and middle doses of ABP: ↑* serum HDL on day 28 High dose of ABP: ↓* serum TG on day 28 ↑* HDL on day 28 - TC and LDL on day 28	(Wu et al., 2020) [37]
1 g BAB extract and 0 g kaempferol/100 g diet/day 0 g BAB extract and 0.08 g kaempferol/100 g diet/day	12 weeks	↓* weight gain		(Kim et al., 2016) [39]
400 mg ABP/kg BW/day	28 days		↓* serum TG - serum TC, LDL, HDL	(Wu et al., 2019) [40]
300 g raw adzuki bean flour/kg BW/day 300 g cooked adzuki bean flour/kg BW/day	8 weeks	Raw adzuki bean flour: - body weight Cooked adzuki bean flour: ↑* body weight	Raw adzuki bean flour: ↓* serum TC and LDL ↑* serum TG Cooked adzuki bean flour: ↓* serum TC	(Zhao, Hou, Laraib, et al., 2021) [10]
500 mg of cellulose/kg of BW/day 5000 mg of cellulose/kg of BW/day 500 mg of EtEx. 40/kg of BW/day 5000 mg of EtEx.40/kg of BW/day	7 weeks 4 weeks 1 week	Weeks 1–7 (500 mg EtEx. 40 or 5000 mg EtEx. 40/day): - body weight	Weeks 1, 4, and 7 (500 mg EtEx. 40 or 5000 mg EtEx. 40/day): - serum TC - TG - phospholipid	(Itoh et al., 2009) [41]
1% of EA 2% of EA	6 weeks	- body weight	- serum TC and LDL ↓* serum TG ↑* serum HDL	(Yao et al., 2014) [44]
1% HWE 5% HWE	13 weeks	↓* body weight ↓* adipocyte size ↓* PPAR*γ*, C/EBP*α*, and AXIN 1 ↑* WNT10B, and DVL2		(Lee et al., 2019) [51]
1% HWE	4 weeks	- body weight gain	↓* serum TC, TG, and non-HDL	(Kitano-Okada et al., 2012) [31]
1% BAB extract 2% BAB extract	12 weeks	↓* body weight ↓* epididymal fat weight		(Yook et al., 2017) [52]
150 g adzuki bean/kg diet/day	12 weeks	↓* body weight ↓* epididymal and perirenal fat weight ↓* adipose cell size - retroperitoneal fat weight	↓* serum TG	(Zhao, Liu, et al., 2022) [32]
100 mg ABE/kg BW/day 200 mg ABE/kg BW/day	4 weeks	200 mg ABE/kg/day: ↓* final body weight and body weight gain 100 mg ABE/kg/day: - final body weight and body weight gain		(Choi et al., 2020) [53]
1 g BAB extract/100 g diet/day 2 g BAB extract/100 g diet/day	8 weeks	1 g BAB extract/100 g/day: - epididymal fat and ↓* body weight 2 g BAB extract/100 g/day: ↓* the epididymal fat and ↓* body weight	1 g BAB extract/100 g/day: ↓* serum TG, TC, HDL, and non-HDL 2 g BAB extract/100 g/day: ↓* serum TG and non-HDL	(Kim, Song, et al., 2015) [54]
6 g AP/100 g diet/day	12 weeks	↓* weight gain ↓* weight ↓* epididymal, perirenal, retroperitoneal, and mesenteric fat weight	↓* serum TG ↓* serum TC ↓* serum LDL - serum HDL	(Zhao, Fu, et al., 2022) [45]
60 mg/kg BW/day of ABTE, ABF, or ABS 300 mg/kg BW/day of ABTE, ABF, or ABS	4 weeks	↓* final body weight ↓* parametrial and perirenal adipose tissue weight	↓* serum TG and TC ↑* serum HDL 60 mg ABTE, 300 mg/kg/day of ABTE, ABF, or ABS only: ↓* serum LDL	(Liu et al., 2017) [48]
15 g GABA/100 g diet/day 25 g GABA/100 g diet/day 35 g GABA/100 g diet/day 35 g adzuki bean/100 g diet/day 0.1 g GABA/kg diet/day 0.1 g Metformin/kg diet/day	6 weeks	↓* body weight	- TC and TG levels	(Jiang et al., 2021) [46]
				(Zhang et al., 2022) [47]
1% freeze-dried BAB powder 0.08% kaempferol	12 weeks	↓* final body weight ↓* the mass and size of epididymal and mesenteric white adipose tissues ↓* *Ppar-γ, Fas, Lpl,* and *Cd36* ↑* *Atgl, Hsl,* ↑*Ppar-α, ↑*Cpt-1α, ↑* *Mcad,* and ↑* *Acox*	↓* TG, TC, LDL, and VLDL	(Kim et al., 2017) [55]
Human study				
44.8 g ABE/day	4 weeks	- body weight - BMI		(Liu et al., 2018) [29]
In vitro study				
250 µg/mL adzuki bean polyphenols 500 µg/mL adzuki bean polyphenols 750 µg/mL adzuki bean polyphenols	48 h	↓* glycerol phosphate dehydrogenase activity in the adipocytes 500 µg/mL and 750 µg/mL adzuki bean polymerized polyphenols only: ↓* glycerol phosphate dehydrogenase activity in the adipocytes	↓* TG concentration in the adipocytes	(Kitano-Okada et al., 2012) [31]
0.5 mg/mL BAB ethanolic extract 1 mg/mL BAB ethanolic extract 1.6 mg/mL BAB ethanolic extract 2 mg/mL BAB ethanolic extract 2.5 mg/mL BAB ethanolic extract 4 mg/mL BAB ethanolic extract	72 h	↓* *C/EBPβ* mRNA expression at hours 24, 48, and 72 when treated with 1 mg BAB extract/mL	↓* TG at early, terminal, and full cell differentiation periods when treated with 1 mg BAB extract/mL	(Kim, Park, et al., 2015) [56]

Triacylglycerol (TG); low-density lipoprotein cholesterol (LDL); high-density lipoprotein (HDL); total cholesterol (TC); ↓ decrease/lower/less; ↑ increase/higher/more; - no change; * *p* < 0.05. Polysaccharides from adzuki beans (ABP); black adzuki bean (BAB); hot water extracts from adzuki beans (EtEx. 40); adzuki bean extract (ABE); extruded adzuki bean extract (EABE); extruded adzuki bean protein (EA); heat-treated adzuki bean protein hydrolysates (AP); GABA-enriched adzuki bean (GABA); flavonoids of adzuki bean (ABF); saponins of adzuki bean (ABS); total extract of adzuki bean (ABTE); hot water extract from adzuki bean (HWE); body weight (BW).

## Data Availability

No new data were created.

## Abbreviations

T2D	Type 2 diabetes
PI3K	Phosphoinositide 3-kinases
AKT	Protein kinase B
HbA1c	Hemoglobin A1C
BW	Body weight
GABA	γ-aminobutyric acid
SOD1	Cu/Zn superoxide dismutase
HOMA-IR	Homeostatic Model Assessment for Insulin Resistance
*Insr*	Insulin receptor
*Irs-1*	Insulin receptor substrate 1
*Pi3k*	Phosphoinositide 3-kinases
*Akt*	*Protein* *kinase B*
*Glut-2*	*Glucose transporter 2*
*Ppar-γ*	Peroxisome proliferator-activated receptor-γ
*C/ebp-α*	CCAAT-enhancer-binding *proteins-*α
*Lpl*	Lipoprotein lipase
*Cd36*	Cluster of differentiation 36
*Atgl*	Adipose triglyceride lipase
*Hsl*	Hormone-sensitive lipase
*Ppar-α*	Peroxisome proliferator-activated receptor-α
*Cpt-1α*	Carnitine palmitoyltransferase 1α
*Mcad*	Medium-chain acyl-CoA dehydrogenase
LDL	Low-density lipoprotein cholesterol
VLDL	Very-low-density lipoprotein cholesterol
TG	Triglyceride/triacylglycerol
HDL	High-density lipoprotein cholesterol
